# A Study on IoT Device Authentication Using Artificial Intelligence

**DOI:** 10.3390/s25185809

**Published:** 2025-09-17

**Authors:** Shahram Miri Kelaniki, Nikos Komninos

**Affiliations:** School of Science and Technology, City St George’s, University of London, London EC1V 0HB, UK; nikos.komninos.1@citystgeorges.ac.uk

**Keywords:** IoT devices, authentication, artificial Intelligence, Internet of Things

## Abstract

Designing reliable authentication mechanisms for IoT devices is increasingly necessary to protect citizens’ private information and data. One of the most significant issues in today’s digital age is authentication. As IoT device technology advances and data grow rapidly, machine learning techniques improve the accuracy and efficiency of authentication and offer advantages over traditional methods, making them valuable in both academia and industry. Device authentication aims to verify legitimate computing devices and identify impostors based on their behavioral data. This paper explores research that applies artificial intelligence algorithms to enhance device authentication mechanisms. We discuss AI authentication models, including deep learning algorithms, convolutional neural networks, and reinforcement learning. We also highlight research challenges and provide recommendations for future studies to support innovation in this field.

## 1. Introduction

There has been a significant technological revolution over the last few years, and the world has begun to witness the onset of this revolution in the realm of the Internet of Things (IoT). This advancement has increased company efficiency and worker productivity while providing enhanced customer experiences. Additionally, it has opened up new business opportunities for creating more innovative products and services.

Both consumers and manufacturers remain concerned about the authenticity of IoT devices. As IoT devices connect to the Internet, they become susceptible to various vulnerabilities. While manufacturers strive to enhance device security, consumers must be aware of the potential risks associated with these devices [1].

An analysis of the IoT highlights its advancements in healthcare, transportation models, and the development of smart cities. The management of network authentication has become increasingly challenging as the number of IoT devices has grown rapidly. Due to its role in device verification, today’s security proposals require device authentication to ensure secure network access [2].

In this crucial moment in IoT development, device proliferation is speeding up, and cyber-attacks are becoming more advanced. Therefore, a survey on AI-based authentication methods is necessary. In this paper, we highlight the weaknesses of traditional approaches and provide a clearer understanding of how AI can uniquely contribute to security, serving as a helpful guide for researchers and industry professionals.

Our paper addresses the challenges related to the use of artificial intelligence in device authentication and discusses solutions and advancements in the field. Our analysis enhances device authentication, making it valuable for researchers and technology vendors seeking guidelines and future directions. Such a comprehensive review not only encapsulates the accumulated knowledge in the domain but also clarifies the potential and limitations of AI in device authentication. This research does not concentrate on non-AI authentication methods, processes that demand substantial computational resources, or studies where security is not the main focus. When we describe something as computationally intensive, we mean processes that require a considerable amount of processing power, memory, or energy, making them unsuitable for resource-limited Internet of Things devices. Examples include large-scale cryptography and complex AI models.

**A. Motivation:**

The goal of this paper is to thoroughly analyze the AI algorithms researchers use to enhance the reliability and security of device authentication. Traditional authentication processes continue to face security risks as computer networks evolve rapidly.

We have identified and categorized various machine learning techniques, along with their applications and effectiveness in real-world settings. Machine learning (ML) algorithms enable systems to analyze data in real time, recognize abnormal behaviors, and promptly implement appropriate responses to emerging security threats. Despite its immense potential, the application of AI in device authentication has not received sufficient attention, as several unresolved research issues remain.

We present promising research opportunities and challenges for device authentication using AI based on the paper’s findings. To achieve our aim, we review advanced device authentication methods with a focus on AI approaches. We compile the existing research on effective device authentication solutions and present our findings along with several unresolved issues for discussion. Lastly, we recommend further investigation in this area based on our findings.

**B. Contribution:**

Our key contributions are as follows:

- Assessing and comparing various AI algorithms and methods to enhance authentication for IoT devices. Additionally, we offer recommendations for authenticating IoT devices.
- Analyzing the strengths and weaknesses of machine learning and deep learning techniques, and outlining scenarios where they could be utilized, along with their accuracy and functionality.
- Outlining current and future research problems in device authentication.

**C. Paper structure:**

The remainder of this paper is organized as follows. Section 2 addresses the security challenges and adversarial threats in IoT device authentication. We examine various types of cyber-attacks that can be executed and vulnerabilities that may compromise IoT device authentication. Section 3 reviews IoT device authentication using AI techniques to understand the security issues and challenges. We also introduce different types of AI and explain the evaluation metrics for authenticating IoT devices.

Section 4 discusses academic research gaps concerning AI-based authentication methods for IoT devices. We explore the limitations of machine and deep learning algorithms, comparing them with traditional authentication techniques and presenting each technique along with its advantages and disadvantages.

Section 5 presents several possible open research areas and challenges in IoT device authentication, with a focus on future research directions. We clarify IoT authentication issues by discussing research results, adversarial techniques, and integration concerns while also recommending research avenues for the next generation of machine learning, deep learning, and reinforcement learning.

Finally, Section 6 concludes the paper and highlights opportunities for the improvement of AI-based device authentication methods.

## 2. Security Challenges and Adversarial Threats

IoT devices face substantial unauthorized access threats due to the absence of standardized protection protocols. Vulnerabilities in network communication and passwords allow attackers to access confidential data. Many vendors neglect updates, resulting in ineffective security measures against attacks. Robust security strategies are crucial for safeguarding data in IoT software environment [3].

Furthermore, adversarial attacks pose significant security risks that target the authentication systems used in IoT devices, as these systems integrate both machine learning and hardware-based components. Vulnerabilities exist throughout the entire spectrum of algorithm structure and software execution. Deep neural networks (DNNs) exhibit considerable susceptibility since attackers can degrade them, leading to misclassification and unauthorized access. The security weaknesses associated with advanced machine learning techniques remain unaddressed due to a lack of response mechanisms to prevent the circumvention of these systems [4,5,6].

### 2.1. Device Authentication Mechanisms

Software vulnerabilities facilitate detection and resolution processes when developers utilize code auditors along with fuzzers, static analyzers, and debuggers. Conversely, hardware vulnerabilities are more challenging to address due to a lack of available tools. IoT manufacturers must remain vigilant to ensure adequate security and safety of their devices [7]. Device authentication involves confirming that a device is legitimate and permits access to a specific area of a network or system. This can be achieved in several ways, including the following:

**Static Authentication**: This is a one-time authentication using credentials such as passwords or certificates. Although it is a straightforward approach, it is vulnerable to specific types of attacks if the credentials are compromised [8,9].

**Dynamic Authentication**: In this method, multiple device authentications occur through the analysis of behavioral and contextual data. It provides higher security by continuously monitoring the device behavior and environment [10,11].

Manufacturers in the IoT industry need to prioritize robust authentication systems that ensure the secure operation of their devices. The advancement of the IoT necessitates changes to authentication systems that must address emerging security challenges and threats [12]. The following two tables were prepared to provide a better understanding of the device authentication mechanisms. Table 1 discusses IoT device vulnerabilities, and Table 2 addresses the IoT device authentication classification.

Table 1 analyzes the risks associated with IoT devices regarding hardware and software vulnerabilities. The articles report various software vulnerabilities, including weak network configurations, a lack of encryption, insecure interfaces, weak authentication, and vulnerable firmware, all of which expose IoT devices to threats such as buffer overflows, data leaks, and command injections [13,14].

In addition to manual reverse engineering, tools such as Firmadyne, DiscovRE, and IoTFuzzer have been used to analyze these vulnerabilities. However, compromises and barriers to mitigation exist due to a lack of resources, the risk of tampering, and the absence of a standard testing framework [14]. Effective security measures include encryption, authentication, hardware security, regular updates, and continuous monitoring [13].

The most common hardware vulnerabilities include weak passwords (default passwords or reused passwords), unpatched components (such as vulnerable TCP/IP stacks), and exposed ports that may be exploited by malware [13,14].

Several tools are available to identify these issues, including Shodan, Nessus, and NIST 800-22. Mitigation is not simple due to resource constraints on many IoT devices (in terms of computing power and memory), as well as the variety of protocols used for communication [17]. Utilizing secure boot, disabling unnecessary ports, and implementing anti-tamper mechanisms can help ensure the security of IoT devices against physical attacks [16].

To prevent adverse impacts on the performance of a device, it is important to address these issues within its limits [15]. In conclusion, this study demonstrates that a security assessment process for every IoT device requires a multi-dimensional approach that balances resource constraints with robust security controls while focusing on the security of each IoT device. It is crucial to continue developing assessment tools and mitigation strategies to enhance the security of IoT devices in the future.

The research summarized in Table 2, which compares IoT device classification authentication, outlines two major approaches to authentication classification: a static authentication approach and a dynamic authentication approach. There are differences between each classification regarding the verification processes, credential types, vulnerabilities, appropriateness, and key technologies. Static authentication, as described in [8,9,18], uses one-time verification with static credentials, such as passwords, pre-shared keys, MD5-hashed passwords, cryptographic keys, or digital certificates. Although these options are simple and have been widely accepted, they exhibit several serious vulnerabilities, including brute-force attacks, password guessing, replay attacks, key theft, side-channel attacks, insecure storage, and phishing.

Moreover, there is a risk of MD5 collusion vulnerabilities. For this reason, static authentication is considered unsuitable for high-security IoT systems and environments susceptible to man-in-the-middle (MITM) attacks, as well as legacy systems that still employ outdated forms of cryptography. The referenced studies indicate that static applications of conventional cryptographic algorithms, as well as static AES/RSA keys, are used but are increasingly inadequate given modern security expectations.

Dynamic authentication is a more advanced strategy based on behavioral and context-based authentication methods as discussed in [10,11,19,20,21]. Typically, dynamic authentication requires a wide range of credentials, including RF fingerprints, sensor fusion data, keystroke dynamics, mouse movements, context information (such as the user’s location), and other device characteristics. With dynamic authentication, identity verification can be performed more flexibly and continuously, especially in environments that require ongoing identity verification.

However, dynamic approaches face challenges, such as inconsistencies in data capture, variations between classes’ behavioral data, and susceptibility to environmental changes. All of which can undermine the trustworthiness of authentication, while sophisticated impersonation attacks continue to be encountered. Nevertheless, dynamic authentication methods offer significant value in high-security Internet of Things (IoT) environments, where continuous user authentication (CUA) is mandatory [11].

For dynamic authentication, machine learning methods (including recurrent neural networks (RNNs), Long Short-Term Memory (LSTM) networks, and deep learning classifiers) and behavioral biometrics are utilized. Both devices and users can be verified continuously and contextually by using these technologies. The integration of Physical Unclonable Functions (PUFs) enhances the reliability and security of dynamic authentication systems. These PUFs provide hardware-based security functions that improve the reliability and security of dynamic authentication systems.

To summarize, the research presented in Table 2 shows that, although static authentication is a useful and simplistic approach, it carries significant risks and is becoming less suitable for securing today’s IoT devices. While more secure and adaptable than static authentication, dynamic authentication can still overwhelm application managers due to data inconsistency, environmental dependencies, and limited resistance to advanced attacks. Further studies related to these issues are necessary to address these concerns.

### 2.2. Threat Landscape in IoT Device Authentication

Unauthorized access to sensitive data or control over device connections poses a critical risk to the IoT devices. Such breaches can compromise privacy, alter data, and even cause physical damage to the system. Standardizing authentication protocols across the IoT ecosystem is challenging due to the wide variety of IoT devices with differing specifications and requirements. Achieving effective authentication of all IoT devices requires a balance between security and usability. There are certain threats that IoT device authentication systems may encounter.

**Spoofing Attacks:** Attacker devices gain unauthorized access by impersonating trusted devices, such as their MAC addresses, IP addresses, or biometric information. They bypass authentication procedures by using stolen credentials or fabricated user identities. Standardized fingerprints and stolen API keys demonstrate how to exploit biometric authorization systems [22].

**Replay Attacks:** Hackers can compromise devices by utilizing valid authentication messages, such as tokens or session IDs, which they steal for unauthorized access [23]. This type of attack involves capturing and retransmitting authentication messages obtained from previous transmissions to deceive the device. For example, an IoT device can be successfully accessed using a stolen OTP or session cookie, illustrating how OTP capture is exploited.

**Adversarial Attacks:** Attackers deceive authentication systems that rely on machine learning models by providing misleading inputs. This leads to erroneous results for ML-based systems such as facial and voice recognition. Manipulating noise in visual data is a strategy that deceives security devices into granting access to unauthorized users [4].

**Man-in-the-Middle (MITM) Attacks:** Attackers position themselves between devices and server connections to intercept and modify the communication flow. These attackers create a point between the device and server to steal passwords or inject harmful data during the authentication process. They obtain login credentials by intercepting data flowing between smart locks and their control applications over unsecured Wi-Fi connections [3].

**Side-Channel Attacks:** Attackers exploit the physical and operational characteristics of a device to expose confidential information through source characterization techniques [22]. They analyze patterns in power consumption, electromagnetic emissions, and timing to uncover encryption keys and other hidden secrets within devices. Using power analysis techniques, attackers can recover a private key from authentication hardware security modules (HSMs).

**Brute Force and Dictionary Attacks:** Attackers attempt to access accounts by using various password guesses, employing both automated, system-wide password tests, and databases of commonly used passwords [24]. They utilize two methods to bypass device credentials, specifically through automated login systems and pre-generated password lists. A botnet system executes thousands of login attempts to breach smart home authentication devices.

**Physical Tampering:** Attackers directly manipulate the device to obtain data, rewrite the firmware, and authenticate without approval [24]. They open the device while performing memory readings, modifying hardware components, and installing malicious firmware. A hacker can access a cryptographic key from an HSM component through physical manipulation during authentication.

**Privilege Escalation:** Attackers exploit system weaknesses to achieve security levels beyond their authorized access. They leverage system vulnerabilities to obtain root access, allowing them to bypass security authentication procedures. For instance, exploiting a buffer overflow vulnerability can provide root access to an IoT authentication device [24].

**Zero-Day Exploits:** Attackers frequently exploit undisclosed vulnerabilities in a company’s authentication system before developers can create patches. When they take advantage of undetected software or firmware weaknesses in devices, they gain unauthorized access through a biometric authentication system, which constitutes vulnerability exploitation [25].

**Social Engineering Attacks:** By using emotional tricks such as fraudulent support phone lines and deceptive emails, attackers gain confidential information from unsuspecting users. These attacks employ psychological manipulation techniques to acquire passwords and authorization access from individuals [24]. For instance, an attacker may convince users to disclose their OTP or password during smart home authentication.

**Firmware and Software Vulnerabilities:** Firmware and software vulnerabilities in IoT devices represent deficiencies in the underlying code that attackers can exploit to compromise the device security. These vulnerabilities allow malicious actors to bypass authorization protocols and gain control over the device, often by injecting malicious code or manipulating input [26]. Such exploits may target individual devices, control hubs, or associated cloud services, thereby leading to significant security breaches. For instance, vulnerabilities in smart lock firmware may enable attackers to disable password authentication through various exploitation techniques, thereby compromising the system’s overall security.

**Denial-of-Service (DoS) Attacks:** Attackers render the authentication system inaccessible by frequently sending an excessive number of requests. These requests can lead to device shutdowns or complete unresponsiveness. By using fake login attempts, attackers generate thousands of requests that prevent the smart home authentication device from recognizing valid users [23]. The impact of these requests makes smart home devices non-functional, rendering them unable to operate effectively. This security vulnerability causes users to face access difficulties and exposes their systems to potential security risk.

**Eavesdropping and Sniffing:** Attackers can access authentication data that is unprotected or poorly encrypted. By monitoring network traffic, they can intercept sensitive security information transmitted through unencrypted HTTP connections that are used to authenticate smart locks [24].

### 2.3. IoT Device Authentication Vulnerabilities and Their Solutions

Several security flaws exist that specifically target authentication methods for IoT devices [27]. IoT devices face challenges in implementing robust security protocols due to functional limitations such as restricted computing power and memory space. Because of these constraints, manufacturers are often compelled to deploy basic authentication systems that cyber attackers can easily compromise [3].

The lack of device monitoring creates significant vulnerability. Manufacturers establish device-specific identifiers, yet many fail to implement security protocols, complicating the tracking of suspicious online behavior. Due to their inability to adopt adequate authentication services that would prevent network threats and attackers from breaching privacy systems, most IoT applications encounter a critical problem [3].

The security of default credentials is crucial because manufacturers often ship devices with pre-existing passwords without advising users to change them. This flaw in authentication mechanisms exposes IoT devices to the risk of unauthorized access. Additionally, organizations can benefit from a wide range of third-party applications available online, although verifying their authenticity poses a frequent challenge. Threat agents may infiltrate the system, compromise the embedded database, and potentially jeopardize the entire system if they install or access unverified applications.

The following measures can be effectively implemented to mitigate the risks associated with IoT device authentication: 1. Encryption can safeguard IoT data from hackers, render them unreadable, and secure communication channels between devices and backends. 2. Security awareness can be enhanced, data breaches and IoT attacks can be minimized, and strong passwords, regular updates, and spam filtering can be encouraged through user guides and training programs. 3. Device monitoring tools and more frequent updates facilitate threat detection and the development of advanced control mechanisms, streamlining processes and protecting devices from major security breaches. 4. The LACKA-IoT is a lightweight access control scheme that adds extra layers of security, aiming to balance the security needs of IoT devices with their resource limitations. 5. Detecting attack patterns in unstructured data through machine learning and deep learning, securing IoT devices, and mitigating emerging threats before they cause significant damage are achievable. 6. Lightweight device authentication schemes are being developed to prevent unauthorized access in resource-constrained IoT environments and to address security challenges as IoT adoption increases.

## 3. Current Research in IoT Device Authentication Using AI Techniques

This section discusses various categories of artificial intelligence, including machine learning and deep learning, that are used to recognize authenticated devices.

### 3.1. Evaluation Metrics

To evaluate AI effectiveness, IoT device authentication systems must use three specific evaluation metrics: the false acceptance rate (FAR), false rejection rate (FRR), and equal error rate (EER), which are essential to ensure the system’s accuracy. The system must effectively differentiate between authorized and unauthorized access attempts. For the authentication system to function correctly, these metrics need to be established; they facilitate access for authorized devices while preventing unauthorized entry, thereby ensuring that the acquired information does not negatively impact users. The purpose of quality measurement methods is to guide in a controlled environment for developers when evaluating and developing authentication models for IoT devices using artificial intelligence. IoT network security requires dynamic adjustment capabilities from AI system implementations that enhance accuracy as well as FAR, FRR, and EER. The evaluation of authentication systems relies on a set of performance metrics, which include

**Accuracy:** The percentage which correct predictions represent

(1) $$\;\mathrm{Accuracy}=\frac{TP+TN}{TP+TN+FP+FN}$$

where we have the following:

- *TP* (True Positives): Legitimate devices were accurately authenticated.
- *TN* (True Negatives): Unauthorized devices were properly rejected.
- *FP* (False Positives): Unauthorized devices were improperly authorized.
- *FN* (False Negatives): Legitimate devices were improperly denied access.

**False acceptance rate (FAR):** The frequency of authenticating unauthorized devices.

(2) $$\;\mathrm{FAR}=\frac{FP}{FP+TN}$$

**False rejection rate (FRR):** The frequency at which legitimate devices are denied access.

(3) $$\;\mathrm{FRR}=\frac{FN}{FN+TP}$$

**Equal error rate (EER):** The point where FAR and FRR intersect indicates the balance of the system.

(4) EER=FAR=FRR

### 3.2. IoT Device Authentication Using ML

Machine learning investigates automated learning processes that enhance performance through experience and produce accurate predictions after analyzing provided data. The nature of machine learning algorithms makes them ideal for passive authentication procedures. The application of machine learning analyzes vast volumes of data to identify validation patterns based on unique device characteristics. Machine learning addresses issues regarding device authentication by enabling scalable, real-time threat detection. The key ML paradigms used include supervised and unsupervised learning.

#### 3.2.1. Supervised Learning

A supervised learning approach involves training artificial intelligence algorithms with labeled datasets to discover hidden patterns between input features and their corresponding outputs [28]. The key objective of the learning process is to develop a predictive model to achieve accurate results when using new real-world data. Some popular examples are regression, vector machine, trees, Bayes, and KNN.

**Linear Regression:** A linear regression model in machine learning employs supervised learning to identify the best-fit line between independent and dependent variables, establishing a linear relationship between them. The authors in [29] present a Trust Management System (TMS) for IoT nodes based on linear regression (LR). It includes simulated datasets in spreadsheet sheets to enhance security in IoT devices, as well as authentication. It simulates a 50-node network containing a database with 5000 entries of five different trust parameters (availability, integrity, security, honesty, and privacy) across 20 iterations.

The TMS was accurate in predicting trust values and detected malicious nodes within two unsuccessful transactions, achieving a 95% threshold (5% error tolerance, α=0.05), and demonstrated confidence in classifying nodes as operational, potentially malicious, or malicious for that environment. Additionally, the TMS required only 1% of the storage space needed by a Neural Network-based TMS (i.e., 100 times less, such as 10 KB instead of 1 MB) and demanded significantly less computational effort, cost, and analysis time.

The authors in [30] focused on the role of ML in power management and optimization for IoT by using regression analysis. The experiments utilized power consumption data collected from a living room air quality monitoring device. This data included various features such as temperature, humidity, occupancy, and the rate of information transmission, among others, along with the actual power usage at different times. The dataset functions both as a tool for environmental sensing and as a part of a continuous authentication system. The methodology developed involved implementing a linear regression method. The research employed Python to perform the data analysis and calculate the coefficients.

The expressed regression equation can be viewed as the following linear regression equation:

(5) $$Y=\beta_{0}+\beta_{1}X_{1}+\beta_{2}X_{2}+\varepsilon$$

where *Y* is the usage power, $\beta_{0}$ is the intercept point, $\beta_{1}$ is the coefficient point of the temperature, $\beta_{2}$ is the coefficient point of the humidity, and ε is the error term.

The regression method enhanced the overall power usage performance and demonstrated that using this method for prediction resulted in a low power consumption. Without this method, power consumption was significantly higher. For instance, the model predicted 145 watts under the specific conditions of 20 °C and 50% humidity.

Linear regression can assist in IoT device authentication. For example, it can serve as a model to predict expected power consumption based on the environment. By modeling expected power consumption and detecting, for instance, tampering or abnormally high or low power usage, this would be a sufficient data-driven authentication method.

**Logistic Regression:** Logistic Regression is a statistical prediction tool and machine learning approach designed for binary classification to determine potential outcomes, despite the name referring to classification rather than regression. In [31], the authors evaluated Logistic Regression (LR) as a supervised algorithm for distinguishing between legitimate and illegitimate IoT devices in smart homes. The experiments utilized datasets derived from network packets captured from real IoT devices, including smart bulbs, smart sensors, and smartphones. These devices are connected to a Raspberry Pi within a smart home network. In the dataset, each row represents a traffic flow, while the columns correspond to feature vectors. The researchers directed network traffic from IoT devices connected via Raspberry Pi to provide LR with information on IP addresses and port specifics. According to the research findings, LR and alternative machine learning approaches are effective in detecting unauthorized devices. Logistic Regression achieved 96% accuracy, 67.8% precision, 80.4% recall, and roughly 73.5% F1-score in the identification of unauthorized IoT devices, which was based on applying the model on 483 network traffic flows analyzed from devices connected through Raspberry Pi, comprising 316 true positives, 150 true negatives, 5 false positives, and 12 false negatives. This combination of accuracy, precision, recall, and F1-score demonstrate the initiative’s ability within the context of this project to provide security to an IoT environment by detecting and preventing the use of non-legitimate IoT devices.

**Support Vector Machines (SVMs):** The Support Vector Machine (SVM) is a supervised machine learning algorithm that creates linear or hyperplane boundaries, separating various classes in an N-dimensional space while maximizing class separation. For example, the authors in [32] studied SVM-based user authentication by analyzing user touch patterns and the movement of mobile devices, and they found varying levels of accuracy. Following this approach, the datasets include multi-touch dynamics and device motion, such as swipe data from touchscreens with capacitive frames and pattern lock unlocking movements; the datasets included some that comprised 160 samples from 8 users. Consequently, along with their work on touch dynamics, swipe data, and user profiling, they indicated that, with high classification accuracy and low error rates, SVMs can serve as viable user authentication mechanisms. Notably, some accuracy levels reached 97.40% and 97.1%, and specific models demonstrated notably low errors with an Average Error Rate of 3.07% and equal error rates of 1–2%. The authors’ findings suggest the practical potential of SVMS as accurate classifiers of user behavior profiles.

**Decision Tree:** The Decision Tree enables non-parametric supervised learning for both regression and classification. It is structured in a sequential hierarchy with a root node, branches, internal nodes, and terminating leaf nodes [33].

In [34], the authors used the Classification and Regression Tree (CART) algorithm for continuous authentication on mobile devices by classifying keystroke events. The public Hand Movement, Orientation, and Grasp (HMOG) dataset was selected for this work because it is publicly accessible and offers extensive data on typing, sensory input, touch, and gestures, making it suitable for mobile continuous authentication. It includes data from 100 participants, with 24 sessions recorded per individual (8 reading, 8 writing, and 8 mapping sessions). This study focuses solely on the 8 writing sessions, which contain 712,418 keystroke events, averaging 327 events per participant. Each keystroke event features detailed information such as press time, intervals between key presses, and key codes. Random samples of unauthorized keystrokes were generated from random events across different users. Finally, balanced training datasets were assembled, comprising roughly equal numbers of known and unauthorized inputs, to simulate typical behavior in continuous authentication, as authorized users are expected to input considerably more data than unauthorized users.

The decision model trained on these features achieved an average accuracy of 0.63 during five-fold cross-validation. While the dataset provides a fundamentally strong basis for evaluating mobile continuous authentication methods, there are limitations. Study participants are demographically homogeneous; their natural usage behavior may differ from the structured, timed sessions of the data collection, and participants were not given contextual explanations for environmental or device (including mobile) use or habits. These limitations restrict the generalizability of the findings, but the HMOG data remains a valuable foundation for comparing machine-learning classifiers’ performance.

Standard ML metrics (accuracy, precision, recall, F1-score, AUC, and MCC) were calculated and analyzed using ANOVA and Tukey’s pairwise comparisons. The CART classifier aligned with the moderate performance of the GBC, RFC, and ETC classifiers (accuracy M = 0.63, SD = 0.05; precision M = 0.68, SD = 0.06; recall M = 0.66, SD = 0.06; F1-score M = 0.67, SD = 0.06; AUC M = 0.61, SD = 0.06; MCC M = 0.41, SD = 0.11). The ANOVA analysis indicated significant differences for all metrics (p < 0.001), and post hoc testing confirmed that for accuracy, recall, F1, AUC, and MCC, CART performed significantly worse than the GBC, RFC, ETC, and k-NN classifiers regarding their ability to differentiate classes.

In [35], the authors implemented Decision Tree (DT) algorithms, including variations like Information Gain and Gini Index, to identify botnet attacks on IoT networks. Both Decision Tree methods accurately classified attack categories with a 0.99 accuracy rate in the Bot-IoT dataset.

The Bot-IoT dataset was created by UNSW Canberra in 2018 and was made publicly available to support machine learning research on detecting botnet attacks on Internet of Things (IoT) devices. The dataset represents IoT devices such as cameras, routers, and printers. These IoT devices were used in a controlled laboratory environment where botnet attacks occurred, so actual data on network traffic was collected. The dataset is divided into two parts: a training set collected from 10 IoT devices over 20 days (with devices identified) and a test set gathered from an additional 9 IoT devices over 7 days (with devices definitely different).

It has two main dependent variables that indicate whether each traffic flow is benign or malicious, and a set of independent variables categorized into traffic flow-based features (number of packets, number of bytes, average packet size, flow duration, ports, protocols) and host-based features (device type, OS version, and manufacturer). These features enabled ML models to be developed that can distinguish between normal network traffic and botnet traffic.

However, the main shortcoming is that it was created in a laboratory setting, which does not capture the complexity and variability found in real IoT networks or the constantly evolving threat landscape. The dataset can be utilized and models can be built accurately, but the results should be interpreted with caution, as models need to be validated against larger, more diverse real-world datasets to be truly generalizable.

Additionally, in [31], the authors employed a Decision Tree to recognize and verify IoT devices in a smart home network. By classifying devices as legitimate or illegitimate based on their network traffic features, the Decision Tree achieved an accuracy of approximately 96.32%.

**Random Forest:** The Random Forest (RF) operates as a machine learning method that accomplishes predictions by employing several Decision Trees to achieve better accuracy and minimize errors [36]. RF has gained popularity as an IoT device authentication improvement technique because it effectively utilizes both network traffic information and device context data. The authors in [37] proposed an RF-based authentication scheme that uses device context, achieving an accuracy of up to 98.1%. The experiments used the context information of IoT devices (identity, activity, GPS location, time zone, and device properties) for 20 to 100 IoT devices, with various-sized context information ranging from 512 bits to 4096 bits. They integrated a Belief–Desire–Intention (BDI) agent with the RF to gather device characteristics before user authentication. This included the validation steps and eventually collecting context device details, device identity, human activity, location, time, and device characteristics. These device characteristics were ultimately transformed into beliefs in the cognitive agency of the authentication server using a BDI model. In contrast, nonlinear regression was utilized by the RF method to create beliefs for authentication, breaking down the votes according to the majority preference, ultimately reflecting the intention to authenticate. The ability of the RF to robustly capture relationships across diverse data and adapt its outcomes led to substantially high accuracy in this context-aware authentication approach.

Based on the research in [35], the authors focused on the accurate and efficient security authentication of IoT devices using machine learning algorithms. It was revealed that the RF algorithm could detect botnet activity in the IoT traffic with 99% accuracy, utilizing the Bot-IoT dataset labeled to train and test the algorithm. While the RF was able to classify malicious IoT traffic with 99% accuracy, the study showed that eXtreme Gradient Boosting (XGBoost) could classify malicious IoT traffic with an accuracy of 99.98% for the attacks being classified, and achieved an attack classification accuracy of 99.99%. Ultimately, XGBoost employs advanced gradient boosting techniques that enable it to better identify and learn from the complex network traffic patterns, thereby enhancing the overall performance in the study.

A study conducted by the authors in [31] examined the use of machine learning algorithms to enhance device authentication in IoT scenarios. As part of this approach, the authors selected the RF algorithm and other supervised learning methods to classify IoT devices. Based on the observed communication patterns, the authors trained the RF model using features extracted from the network traffic data. By distinguishing and confirming devices, the goal was to improve the security of IoT networks.

In [38], the authors presented the RADTEC framework, which achieves over 95% accuracy and a time of less than 0.65 ms by using machine learning to classify IoT device types based on measurable data found in packet headers. RADTEC relies on network traffic data (packet capture (pcap) files) from the University of New South Wales and includes traffic from over 28 active (15 selected) IoT devices. First, the framework detects and analyzes critical fields from network flows and creates a device fingerprint upon completing the adjustments. The device fingerprint is classified using fast machine learning models, primarily the Random Forest, due to its high accuracy and low latency. The RF, based on the authors’ deep learning study, has the highest accuracy and throughput together, allowing for optional iterative classification to improve accuracy and consistency. With the efficiency and accuracy of device classification, the RADTEC framework enables continuous and real-time device authentication. According to the study, RF is essential in enhancing the security and reliability of IoT device authentication systems.

The authors of [34] conducted a comprehensive evaluation to understand how machine learning classifiers function for continuous authentication (CAauth) on mobile devices when detecting keystroke dynamics. The researchers concluded that ensemble algorithms achieve their best results by utilizing Random Forest after conducting their analysis. The analysis of the RFC for the 100 HMOG dataset users generated these performance metrics: an accuracy rate of 0.68, a precision of 0.71, a recall of 0.76, an F1-score of 0.73, and an AUC of 0.72, while MCC amounted to 0.59.

The authors in [39] took advantage of the unique human gait characteristics that were utilized in this study for the continuous authentication of remote IoT users from both mobile phones and wearable sensors. The authors captured gait activity from 30 users in the age range of 15–34 at the time of use, with each subject given a Samsung S7 Edge smartphone to use that used an Android application to capture both the accelerometer and gyroscope sensors at a 50 Hz sampling rate.

For authentication, the authors utilized features from 10 gait cycles per user. The authors applied machine learning techniques; Random Forest provided better performance than the other algorithms used in this research, and thus, it was employed as the classifier.

The authors created a 70/30 train–test split of their data and then used 10-fold cross-validation, achieving 94% authentication accuracy and an equal error rate (EER) of 6% with their framework. This type of authentication offers accuracy, unobtrusiveness, and continuous authentication on IoT devices while also ensuring sufficient security and privacy without requiring user input on resource-constrained devices.

The authors in [40] outlined research aimed at discovering unauthorized Internet of Things (IoT) devices on organizational networks using a machine learning model. The research team collected and tagged vast amounts of TCP/IP traffic data from 17 different IoT devices of nine types over a period of multiple months in two laboratory settings. Having collected the above data, the team trained a Random Forest using a white-listing style with a majority vote approach on each device type during 20 sessions to enhance overall accuracy.

The sampling of 17 devices resulted in a high accuracy of detecting unauthorized IoT device types (96% on average) and white-listed device types (99% on average). The research also measured the time it took for devices to be detected (some within five TCP sessions, with 100% detection achieved by 110 sessions). Additionally, it demonstrated that the classifier performed well in various lab settings while also being resilient against attacks.

**Nearest Neighbors (KNN):** The K-Nearest Neighbors (KNN) algorithm is a supervised learning classifier that employs non-parametric methods to forecast individual group associations through distance-based proximity calculations. It is one of the most popular and simplest classification and regression classifiers used in machine learning today.

The authors of [32] studied the integration of machine learning algorithms into user authentication schemes. To do this, they collected data on 30 unlocking gestures, implementing the KNN algorithm for classification. The authentication performance using KNN was evaluated using the equal error rate (EER) metric. The KNN algorithm obtained an EER of 4.90% in relation to a touch and device movement-based authentication scheme.

In [34], the authors discussed a k-NN based on key-pressing dynamics for continuous authentication, which achieved an average accuracy of 65% during training and testing using the HMOG dataset. The details of these models relate to the keystroke dynamics model on which they are based. Keystroke features include the keys pressed and the time between key presses. Additionally, a balanced dataset of 100 users was created, containing both authorized and unauthorized key press events. Finally, five-fold cross-validation was employed. Thus, the accuracy, or number of correct predictions, was computed.

In [41], the authors also studied the effectiveness of learning algorithms for keystroke-based user authentication. The KNN algorithm achieved 74.58% accuracy for authenticating genuine users and 98.61% accuracy for detecting an impostor using the CMU Keystroke Dynamics Benchmark Dataset, which consisted of keystroke typing data provided by 51 user examined features were dwell time, flight time, and latency time for digraphs. The dataset was then split such that the first 300 rows of typing were assigned as a training set for each user. At the same time, the last 100 rows of typing belonged to the original user, and 100 rows were randomly selected from unrelated users and verified as impostors or not. The artificial neural applications were then implemented to classify these as real or impostor classifications for evaluation.

**Naive Bayes:** The Naïve Bayes algorithm is a probabilistic machine learning algorithm that performs classification operations based on Bayes’ Theorem. The model achieved computational efficiency through conditionally independent assumptions when applied to real-world scenarios. According to [32], the Gaussian Naive Bayes (GNB) employs purely behavioral biometrics (all users had the same pattern) and attained 95–97% accuracy with touch-based pattern lock authentication, establishing it as the best and most efficient algorithm while also examining accuracy across multiple postures.

An average accuracy of 0.64 was reported in another study on continuous authentication using keystroke dynamics [34], indicating that Naive Bayes may not be suitable for such data. In detecting botnet attacks, Naive Bayes achieved a high accuracy of 0.99 in IoT security, but it showed reduced performance of 0.71 when classifying the attack type. The use of GNB was implemented in another study [31] to validate smart home IoT devices, resulting in a 74% accuracy rate based on network traffic analysis. Naive Bayes proves effective in various security contexts according to these studies, but its performance varies depending on the dataset and application.

Table 3 discusses several advantages, disadvantages, and security concerns for IoT device authentication using supervised machine learning algorithms.

Table 3 provides an overview of the various supervised machine learning (ML) methods used to authenticate IoT devices. The precision and computational performance of all methods are examined and documented in each analysis, along with their security weaknesses. This information delves deeper into the contributions of these studies: To authenticate IoT, it is essential to identify complex relationships with a high accuracy rate of 97.1% as shown by SVMs [32]. This accuracy results from the effectiveness of the SVM in distinguishing between classes in high-dimensional spaces.

To maintain performance levels [32], the method requires substantial computational power and a large number of training datasets. For instance, SVM training takes 5–10 min for 1000 samples and 2 h for 10,000 samples. SVM models face accuracy limitations due to their vulnerability to adversarial attacks, thus producing false rejection rates that can reach 50% when trained on restricted datasets, according to [28,32]. Dynamic data patterns and evolving threats pose significant risks in IoT applications in real-world settings. The results from RF vary across different applications in various studies, such as 68% for keystroke recognition in HMOG interactions [34], 98.1% for the authentication of IoT devices [37], 99% for detecting botnets with the BoT-IoT dataset [35], and 95.2% for IoT re-authentication in UNSW [38]. Although RF is remarkably precise, many devices cannot utilize it due to the significant memory and processing power required to handle large datasets; for example, 16GB RAM is needed to process 30K rows with 500 features and 1000 trees, and the imbalance in RF models exacerbates this issue [32,38]. These security concerns are associated with susceptibility to attacks via adversarial relaying inputs, such as denial-of-service (DoS) attacks, or even alterations to the training data [31,35,37].

An IoT ecosystem would suffer tremendously from all these issues, which would undermine the model’s defenses. KNN has been studied for various IoT-enabled authentication tasks, achieving 74.58% accuracy in keystroke authentication [41] and showing equal error rates (EERs) of 2.52% for familiar users and 4.90% for gesture-based authentication processes [32]. Although KNN excels in these scenarios, its use entails a high cost in computational processing, especially when dealing with large datasets like the HMOG dataset, which consists of 712,418 keystroke events [34]. Moreover, KNN has a 25% false rejection rate (FRR) for authentic users in noisy contexts [41], meaning that spoofers manage to attain false acceptance rates (FAR) of about 10% and 15% [41]. Privacy concerns, along with the data variability of KNN-based models, make them unreliable for mobile IoT applications, according to [32,34].

Naïve Bayes (NB) is a reliable and simple tool capable of achieving accuracy rates of 64% [35], 74.38% [34], and up to 99% in specific IoT tasks [31]. The accuracy reduction reaches 64% in complex situations where NB models demonstrate independence, although it provides advantages according to research [35]. Furthermore, NB models are at risk of misclassification as indicated by a Matthews correlation coefficient (MCC) of 0.45, which shows that they have a moderate level of classification performance [32,35]. The maximum vulnerability of the NB model due to data manipulation diminishes the reliability of these models in adversarial environments, raising security issues.

Decision Trees (DTs) have been highly successful in addressing a significant number of Internet of Things (IoT)-related problems, achieving a classification accuracy of 96.32% when applied to IoT devices in smart homes [31]. Additionally, an accuracy of 0.63 has been achieved through the use of keystroke-based data in mobile context-aware (CA) applications [34]. In contrast, an accuracy of 99% has been found in IoT botnet detection [35]. Mobile computing activities utilizing DT models show varying accuracy rates between 0.55 and 0.86, depending on the volume of data and selected features [34]. DT models have demonstrated high accuracy (0.97–0.98) in IoT behavior but fall short compared to the performance of advanced algorithms like XGBoost [35]. Security threats are characterized by their vulnerability to keystroke pattern eavesdropping in mobile context-aware activities [34], as well as traffic disruption from IoT botnet attacks [35]. A relatively rare risk of misclassification arises when the training dataset comprises a homogeneous dataset, leading to elevated false negative and false positive rates [31].

Logistic Regression (LR) demonstrates sufficient accuracy in small Internet of Things (IoT) applications when dealing with constrained data collections. LR has been effective in predicting 483 traffic flows [31] and exhibits applicability on devices ranging from 2 to 8 on a Raspberry Pi board [31]. However, LR is less effective than Decision Tree (DT) models, which achieve an accuracy level of 96.32% [31]. Moreover, LR does not perform well under conditions of small sample sizes, restricting its applicability to many IoT settings [31]. Its security flaws include susceptibility to packet tampering attacks and excessive false positive and false negative rates, with risks of 5 false positives and 12 false negatives for access control configurations [31].

Linear regression has also been used to estimate electricity consumption in boarding houses in [42], based on the (Rooms 9A, 10A, and 14A) datasets collected every minute over two to four months. Five independent variables—voltage, current, power, frequency, and power factor—have been employed to estimate electrical energy consumption. The approach achieved high real-time accuracy of 98.07%, with low latency (<3 s), and efficient billing estimation at 91.98% accuracy, along with an RMSE of 0.0493, demonstrating timely and reliable energy prediction. Despite these advantages, linear regression has several limitations. It can only predict day-based intervals, restricting hourly or dynamic forecasts. Variable prediction performs well in some cases, such as 89.48% accuracy in Room 10A with an RMSE of 0.0596, but overall flexibility remains limited. Security concerns are also significant, with faulty or default passwords on the PZEM-004T and NodeMCU, as well as insecure APIs, which potentially compromise system integrity [42].

All ML methods are valuable for IoT device authentication but face significant computational, performance, and security challenges. Their vulnerability to attacks, data tampering, and IoT threats requires developing robust, adaptive authentication systems to adequately protect IoT environments.

#### 3.2.2. Unsupervised Learning

Unsupervised learning functions, as a machine learning method, extract information from samples of untagged data. An unsupervised learning model manages unlabeled data by discovering patterns, as it does not receive guidance or direction from a supervised approach [28]. Clustering and Principal Component Analysis are some examples.

**Clustering Algorithms (k-means/Hierarchical clustering):**

K-means and hierarchical clustering often employ unsupervised machine learning algorithms to group similar data points into distinct clusters. K-means partitions data into k-exclusive clusters by calculating the distances to centroids, whereas hierarchical clustering generates a cluster hierarchy structure using either divisive or agglomerative approaches [28].

The authors in [43] proposed a hybrid security framework for IoT network devices based on ML and K-means clustering for intrusion detection. The framework was developed using Object-Oriented Analysis and Design Methodology and the SQLite database management system, and has been designed to address the security issues facing IoT devices by collecting data from the devices and monitoring network traffic baselines.

K-means clustering was used to understand normal behavior based on characteristics of the device as well as to prepare the data by clustering like data points together, to group patterns for anomaly identification, and to reduce the dimensions of the information to allow scaling. An unspecified ML model performed anomaly assessment using a varied set for each cluster; as the documents were re-clustered, the training on the models would be updated. The proposed approach is based on clustering and ML for intrusion and anomaly detection after the data has been collected. The results of the evaluation showed 87% of detection of known intrusions with a 15% increase in the identification of unknown threats over previous versions of the method, with a false positive rate of 8%. However, there are issues with finding known intrusions, resulting in a 5% decrease in accuracy due to the rapid shifting of K-means traffic; an increase of 20% in processing time allowed for heterogeneous devices.

**Principal Component Analysis (PCA):**

PCA is a machine learning technique that converts high-dimensional data to low-dimensional spaces while preserving meaningful information for better data analysis and modeling tasks [44].

According to [45], the authors discussed that PCA has also been utilized in previous ML-based PIN entry system attacks on smartphones. In these attacks, PCA was employed to process WiFi Channel State Information (CSI) data recorded during Personal Identification Number (PIN) entry, thus reducing data dimensions while keeping the most discriminative features corresponding to keystrokes. Authenticators successfully recovered PINs because the dimensionality reduction technique extracted critical signal features from WiFi communications, revealing major weaknesses in traditional authorization frameworks. The PCA-based inference vulnerability highlights the crucial need for improved security procedures.

Table 4 demonstrates an evaluation of unsupervised ML authentication methods for IoT devices, detailing their benefits, drawbacks, and security challenges. A thorough assessment of authentication systems using K-means clustering and Principal Component Analysis (PCA) draws from the available empirical evidence in the current academic literature. K-means clustering employs preprocessing methods to enhance authentication accuracy, achieving a tested purity gain of 0.877 compared to the baseline of 0.44. This improvement is beneficial for differentiating between authorized and unauthorized devices as specified in [41].

Additionally, the algorithm has been shown to classify data into clusters of sizes ranging from 2 to 50 members, making it applicable to a wide range of Internet of Things (IoT) authentication systems [46]. Despite this, one of the main drawbacks of K-means clustering is its noise sensitivity, which can significantly destabilize classification. The study demonstrates that in the case of keystroke authentication, the purity may be as low as 0.44 when there is no preprocessing; hence, data augmentation is required to achieve effective classification [41].

Furthermore, while K-means clustering is utilized, prior knowledge of the number of clusters (k) is necessary, which limits its effectiveness in dynamic IoT settings with changing device usage and network conditions [46]. Regarding security matters, K-means clustering is vulnerable to denial-of-service (DoS) attacks, as invasive noise can adversely affect the performance of the clustering operation and lead to erroneous authentication outcomes [46]. This vulnerability is expected to result in the unintentional rejection or incorrect acceptance of legitimate devices, thus representing a severe security threat to Internet of Things (IoT) frameworks [41].

Authentication systems based on the IoT benefit from Principal Component Analysis (PCA) as a common technique to reduce their data dimensionality and enhance computational performance. Research findings indicate that analytical results improve after reducing data dimensions and processing time by applying PCA [41].

However, empirical research shows that PCA has also been utilized in keystroke inference attacks since it can compress CSI data, enabling the attack to distinguish keystrokes with varying degrees of accuracy ranging from 64% to 82% [45].

This feature poses a significant privacy threat, as it makes it easy for unauthorized parties to infer a victim’s personal data. Even though PCA excels in feature extraction, it performs poorly in clustering for keystroke-based authentication as evidenced by a negative silhouette value and a purity of 0.20, which together indicate a lack of enhancement in classification accuracy [41].

Additionally, PCA is highly vulnerable to variations in adversarial sets. Adversarial perturbations in the input can influence the derived principal components, thereby undermining the reliability of authentication systems [41]. Furthermore, another drawback is its limited ability to detect slight variations in Channel State Information (CSI) measurements, particularly concerning complex hand and finger movements, which degrades classification performance [45]. The security risks associated with using PCA for keystroke inference attacks stem from inherent weaknesses. Attackers enhance their ability to gather and identify classified user information through the use of dimensionality reduction techniques [45].

When preprocessing steps are implemented, K-means clustering demonstrates improved accuracy. However, it has significant issues with noise attacks and denial-of-service attacks, rendering the system less reliable in challenging conditions. PCA effectively reduces the feature dimensions while simultaneously exposing serious privacy vulnerabilities through keystroke inference attacks. This table highlights the need for stronger protective measures to address potential weaknesses in machine learning authentication systems in the future.

### 3.3. Reinforcement Learning (RL)

Reinforcement learning (RL) is a key technology for enhancing IoT device authentication due to its adaptive learning capabilities. By utilizing RL algorithms, systems gain knowledge through interactions with the environment, which autonomously improves security mechanisms over time [47]. RL enables a response to evolving IoT threats, allowing for better adaptation to dynamic security challenges.

The authors in [48] developed a 3D geometry channel model for RSMA-IRS-assisted ISAC systems in an urban environment. The 3D model describes LoS/NLoS fading, Doppler effects with user mobility at 1 m/s and target mobility at 5 m/s, and radar cross-section (RCS) modeling for targets up to 20 m^2^. They formulated an energy-efficiency maximization problem under the same configuration, allowing beamforming and IRS phase-only adjustment with two quantization bits, while the system must meet QoS (minimum rate of 4 bit/s/Hz) and radar SNR requirements (0 dB). This is solved with a PPO-based deep reinforcement learning framework, where the state space includes SINR values for both users, Doppler shift data, and radar echo data; the reward function promotes QoS but penalizes when QoS is not achieved or when satisfactory SNR is not reported (e.g., $\Omega_{\mathrm{QoS}}$ drops to zero when QoS is unmet).

The authors conducted simulations with 2 users, 4 BS antennas, 9 IRS elements, and a carrier frequency of 2.4 GHz (adjusted to 1.4 GHz). They reported that after 1 million iterations, the agent converged relatively quickly, achieving energy-efficiency improvements of up to 50% compared to SDMA and a 67% EE reduction compared to double-Rayleigh fading at higher frequencies. In SAGIN/RIS networks, for IoT device authentication, robust capabilities support Doppler-based frequency shifts with a vehicle speed of 1 m/s and radar-echo properties with 0 dB SNR thresholds, based on multiple SINR policies as unique fingerprints involving numerous possible iterations up to $10^{6}$. The DRL framework enables recognizing authentication by embedding penalties for spoofing, e.g., $\Omega_{\mathrm{Echo}}=0$ when SNRs are outside similarity thresholds, and using adaptive beamforming to differentiate legitimate signals from 2-user interference, providing a self-sufficient, secure, and energy-efficient authentication method that is difficult to spoof in high-mobility scenarios without solely relying on cryptography.

According to [28], RL is effective for real-time anomaly detection, as it identifies unusual patterns that may indicate potential security attacks. Moreover, the authors in [28] stated that RL outperforms traditional ML models in detecting malware on IoT devices due to its inherent adaptability. RL-based authentication can be implemented as devices learn from their environment without requiring prior training data.

Additionally, RL can be applied in intrusion detection systems (IDSs) and adaptive honeypots, both of which can utilize this technology to defend against attacks and malicious behavior. Furthermore, RL can assist with interoperability by determining how to communicate with poorly documented devices [47].

Table 5 provides a side-by-side comparison of reinforcement learning-based authentication in IoT devices, explaining their merits as well as the specific deficiencies and security concerns that each poses as the IoT environment becomes more heterogeneous and dynamic. A study by [49] examined the effectiveness of Dynamic Q learning with the Double Estimation Strategy (DES DRL) for changing authentication challenges based on context-related risks as they arise. Based on the G-Mean, the approach is highly accurate, with a specificity of 92.62% for categorizing authentication requests, while the DES DRL captures most of the true positives as well. To adapt to changing threat scenarios, the system is designed to retrain every 1000 observations. However, the system requires a substantial computing resources [49] since offline training takes approximately 130 s and convergence demands around 6000 samples (one week). With a factor of 0.25 and a λ value set to 1, the model still displays susceptibility to familiar threats, particularly from trusted users such as coworkers. To maintain privacy, data processing is performed directly on the device, thereby reducing the risk of exposure to sensitive information.

In [47], the authors investigated the use of real-time learning for optimizing IoT device interaction sequences. The system aimed to achieve Goal 1 in two steps and Goal 2 in four steps after 400 interactions. Although the approach converges quickly to more complex goals in some situations, it becomes more complicated as more commands are added (approximately 100–600 commands are required to reach Goal 2). Moreover, the system operates under a rate limit, resulting in delays of about 40 min for every 100 episodes. Among the main security issues identified are the interoperability of poorly documented protocols and the risk of adversaries exploiting the learned state machine to manipulate protocol states maliciously.

In [50], the authors presented a hybrid deep learning (DL) and reinforcement learning (RL)-based authentication framework designed for use in heterogeneous IoT environments. Several experiments have demonstrated that the model supports a wide range of IoT applications with high accuracy and effectiveness. However, this method lacks generalization to real-world scenarios and specific metrics for evaluating accuracy that are necessary for practical implementation. In addition to data integrity threats (e.g., data tampering), device heterogeneity creates significant authentication challenges. Furthermore, the increased complexity introduced by the model processing mechanisms heightens the potential for DoS attacks.

In [51], the authors developed an adaptive ε-greedy RL approach that updates the exploration–exploitation parameter (ε) based on the volume of observed attacks. In terms of the packet delivery ratio (PDR), the system can successfully handle both static and dynamic data sources, achieving a PDR of 1.0 for non-traffic and 0.929 for malicious traffic at 160 units. Although its end-to-end (E2E) delay increased to 1489.474 ms for malicious traffic at 40 units, it only increased to 1177.795 ms for normal traffic. This delay may adversely affect the time-critical IoT applications. A proxy user attack occurs when a third party exploits a secure user identity and impersonates an entity. In contrast, a black hole attack involves malignant nodes dropping packets at the network layer. Additionally, IoT devices have limited memory and computational capacity, making the processing of high attack volumes particularly challenging.

According to [52], a hybrid RL model is presented to address internal threats by using elliptic curve cryptography (ECC) the and Lightweight Directory Access Protocol (LDAP). By using the nonces $r_{1}$ and $r_{2}$, the system ensures that no plain text data is exchanged, which maintains data confidentiality. Although the setup is robust, the authentication process is expensive, taking more than 72 h to authenticate 1000 users in a Jupyter notebook with the parameter choices (α=0.1–0.5 and γ=0.6–0.9). IoT devices with resource constraints are not suitable for this type of system. Even if the nonces are exposed in some way, the model still provides a high level of security, though man-in-the-middle attacks are not impossible. In addition, if the LDAP or ECC fails, the attacker remains an insider, and certain vulnerabilities may go unaddressed, such as spoofing and replay attacks.

In combination, the studies reported in Table 5 illustrate the potential of RL for IoT device authentication by providing solutions that are flexible, data driven, and responsive to emerging security threats. It is important to note that the proposed implementations have some limitations as well—for instance, they use expensive computation curves, have relatively slow response times, and are not foolproof against advanced attacks. Based on these limitations, additional research is required to improve these approaches for the practical deployment of resource-constrained IoT systems. To fully leverage RL-based IoT authentication systems, it will be crucial to keep pace with advancements in RL and security. The hope and challenges for enhancing IoT authentication systems are highlighted by RL.

### 3.4. IoT Device Authentication Using DL

Deep learning (DL) algorithms employ a multi-level neural network that uses numerous nonlinear processing layers so that the representation of the data learned is learned on the basis of the use of layers determined by a deep learning procedure to find patterns of any data outputs. DL approaches are noted to be a robust method for many contexts in image recognition to categorize images for convolutional neural networks (CNNs), general classification tasks for artificial neural networks (ANNs), and for sequential data such as speech and text for recurrent neural networks (RNNs). The ability of DL techniques to learn complexity makes them suitable for IoT systems due to the volume of data and the advanced representation of data on a global scale, and now we are beginning to see improvements around the complex representation of data to help in the security of IoT systems including authentication [53].

**Neural Networks:** Neural networks consisting of interconnected neurons are an effective tool for authenticating IoT devices. These networks process and analyze data, recognizing patterns and making decisions based on input. By adjusting connections and weights, neural networks learn from data and improve performance over time, making them particularly useful for verifying device identity in resource-constrained environments.

Neural networks can acquire data by inspecting radio frequency signals and analyzing device operability to distinguish between legitimate devices and security threats, according to [54]. They demonstrate significant capabilities in maintaining IoT network integrity due to their adaptability and learning potential. In [54], the authors proposed a unique authentication method for remote wireless devices based on self-organizing feature maps (SOFMs), a type of neural network designed to characterize RF fingerprint signatures.

To collect raw RF data, they built an experimental testbed that satisfies the essential requirements for IoT device authentication, particularly among the less secure, low-cost, and long-range technologies in use today, such as LoRa. A unique SOFM algorithm was employed to preprocess the RF data and interpret the highly correlated signals into real-time RF fingerprint patterns. To determine the actual classification and authentication of each device, they integrated those patterns into CNNs. The results of their study showed nearly 100 per cent accuracy in identifying LoRa devices at an individual device level using a standard PC CPU; therefore, the novel method demonstrated considerable computational efficiency, leading to significant improvements in RF cyber–physical security.

The authors in [55] proposed a Process-based Pattern Authentication (PPA) method to improve the security of Internet of Things (IoT) devices by using dynamic pattern generation for authentication and touch pattern modeling with the help of an ANN network. Specific authentication patterns for each login session are created during the PPA process by combining user-input information (R-code) and the server-generated challenge (P-code), resulting in a pass-code.

The ANN performs touch dynamics analysis by measuring pressure and velocity parameters to achieve accurate user identification and authentication. It is trained on a database of 29,008 samples from 35 users, reaching a classification accuracy of 99.75%, a false rejection rate (FRR) of 5.03%, and a false acceptance rate (FAR) of 4.36%. Capable of preventing attacks such as shoulder surfing and smudge attacks, the PPA system provides a highly secure environment for IoT devices.

**CNN: Convolutional Neural Networks**

CNNs function as deep learning algorithms that utilize multiple processing layers to learn data representations and analyze patterns. They employ sparse interactions, parameter sharing, and equivariant representations to decrease the number of data parameters compared to traditional artificial neural networks (ANNs). CNN architectures vary, consisting of cascading convolutional and pooling layers organized with multiple filters for convolving data parameters. The pooling layers typically perform down-sampling, resulting in smaller subsequent layers that may use maximum pooling or average pooling across a range of layers. Internally, the features include a key component called the activation unit, also known as the activation function, which applies a nonlinear activation operation—most commonly the rectified linear unit (ReLU)—to the features [53].

In [56], the authors used a convolutional neural network (CNN) to improve physical layer authentication in wireless communications.

The experiments used a dataset (4000×256) of RSS behavior, comprising 2000 channel recordings from Alice to Bob and 2000 from Eve to Bob, collected with USRP devices in a conference room measuring 6m×4m. Specifically, CNN depends on a Data-Adaptive Matrix (DAM) that incorporates channel statistics that change over time. It consists of two convolutional layers with 2×2 kernels and ReLU activation, two max-pooling layers with 2×2 kernels, and a final fully connected layer with a logistic activation function for classification. The detection rate of the CNN was 100% when SNRs were 6 dB and higher, and 95.89% when the SNR was 4 dB. Research findings show that the CNN yields superior results compared to GMM and SVM in detecting spoofing attacks in dynamic system environments.

The authors in [57] discussed EENet-Lite, a lightweight early-exit CNN that uses whuGAIT IoT data and incorporates authentication methods based on gait recognition for IoT devices. The model features early-exit branches and specialized loss functions to balance accuracy and efficiency. It achieves an accuracy of over 85.00% while reducing multiplications, additions, and relational operations (MAC) by a factor of 5.9 compared to traditional deep neural networks (DNNs).

Additionally, the model supports intermittent computing through checkpointing, which enables it to save up to 34% of redundant computations. EENet-Lite also has between 166.67- and 357-times fewer parameters than ResNet-based models, making it well-suited for deployment on low-power platforms with limited memory.

The study in [58] described a new IoT authentication mechanism based on EEG signals (via a NeuroSky MindWave headset) and hand gestures (via a lightweight CNN) to meet one of the requirements of 92% effectiveness and 93% efficiency involving 30 subjects. The EEG signals are processed to determine a binary based on the levels of attention and meditation over time, using adaptive thresholds, and can generate up to 200 possible values for each bit.

For the hand gestures, we define three gestures: closed hand, open hand, and raised index finger. In total, there are four states related to the authentication process, each involving one of the hand gestures and the transitions between them, all implemented on a Raspberry Pi. The system achieves user satisfaction deemed acceptable based on the satisfaction assessment, with an average authentication time of 33 s when measuring a 4-bit key.

The security analysis indicated that the 4-bit EEG password was 4.3 times stronger than a 4-symbol ASCII password and that EEG signals could resist physical observation and impersonation threats. The work demonstrates that deep learning (CNN) can be used as a method for gesture recognition with IoT devices in a way that adheres to compatibility standards for authentication mechanisms as a security priority.

**RNN: Recurrent Neural Networks**

Recurrent neural networks (RNNs) are a class of deep learning algorithms developed to work with sequences of data. The prediction in these neural networks relies on current and past inputs. RNNs have a time layer that encodes temporal data; therefore, they can learn complex changes in their recurrent hidden units [53].

In [59], the authors developed an ECG-based authentication system for IoT devices using a deep recurrent neural network (DRNN) architecture, which applied a bidirectional and late fusion approach. The data to be authenticated in this study are ECG signals, which they processed with derivative and moving average filters. They segmented the ECG data using the detected R-peaks to create fixed-length input windows for real-time performance.

They evaluated their model using two open datasets, the MIT-BIH Normal Sinus Rhythm Database (NSRDB) and the MIT-BIH Arrhythmia Database (MITDB). The authors reported 100% precision, 100% recall, 100% accuracy, and an F1-score of 1.0 from NSRDB; from MITDB, they reported 99.8% precision, 99.8% recall, 99.8% accuracy, and an F1-score of 0.99. The authors demonstrated that the DRNN has high efficacy and reliability in delivering accurate and efficient real-time authentication in the IoT context.

The research in [60] presented an RNN-based model for anomaly detection in UAV sensor data that classified a pavement with 99.7% accuracy in detecting anomalies in north speed and up to 100% for pneumatic lifting speed anomalies. The analysis was based on real UAV flight data, with 60% used for training and the remaining 40% for testing. The model was trained solely on normal data to identify anomalies with 99% confidence.

The north speed had a false negative rate of 7.7%, and pneumatic lifting had a false negative rate of 0.0%, with neither showing any false positives. Overall, these results demonstrated that the model performed well and offered strong extrapolation. Furthermore, it presents an intelligent model based on time-series data that could be utilized in behavioral authentication within IoT-based systems using RNN architectures.

**LSTM: Long Short-Term Memory network**

The Long Short-Term Memory (LSTM) network uses a recurrent neural network structure to solve the gradient vanishing problem and improve its ability to learn sequential patterns in data. LSTMs are vital in enhancing the security and dependability of IoT systems by offering strong methods for detecting and identifying rogue or compromised devices.

The research studies [53,61] demonstrate the critical role of recurrent neural networks (RNNs), especially Long Short-Term Memory (LSTM) networks, in improving Internet of Things (IoT) security through advanced authentication techniques. In source [53], the authors discussed how LSTMs are used in network traffic analysis to detect malicious activity by accurately classifying network flows, highlighting their potential in real-time threat detection. Conversely, the authors in [61] presented an LSTM-based classifier in the IoT gateway for authenticating device-originated signals and defending against data injection attacks. Their method achieved high detection accuracy with minimal latency and processing costs as shown through simulations modeling LoRa transmitters and embedded watermarks. The flexibility of LSTMs is clear from these outcomes, as they deliver IoT security solutions both at the network and device levels, forming an integrated defense system.

The authors in [62] employed the LSTM deep learning technique to predict security attacks targeting MQTT-based Internet of Things (IoT) networks. The KDDCUP99 MQTT dataset was used to train the model with various attack types, including DDOS, DoS, Bot, BruteForce, and Infiltration.

The KDDCUP99 MQTT dataset was chosen because it is one of the few large-scale datasets specifically adapted for IoT environments. Unlike original datasets, such as the KDDCUP99, which are not designed for any particular IoT protocols or traffic patterns, this dataset includes over 10.7 million instances of IoT device data and remains representative of real-world environments (e.g., smart homes, smart cities, industrial zones, and automated systems). It contains both benign traffic and a range of modern cybersecurity threats (e.g., DDoS using HOIC and LOIC-HTTP, DoS with Hulk, SlowHTTPTest, and GoldenEye, botnets, brute force attacks on FTP and SSH, and infiltration attacks), making the KDDCUP99 MQTT dataset more advantageous than other datasets that fail to capture IoT traffic characteristics. However, a significant limitation is the imbalance between benign traffic and attack classes. Although the dataset provides extensive data, this imbalance inflates accuracy in benign traffic prediction and leads to the frequent misclassification of attack classes. Moreover, this limitation impacts our conclusions regarding the challenges of imbalanced datasets in IoT security. It also underscores the importance of employing additional techniques, such as GloVe embeddings, and highlights the need for more datasets that are balanced and reflective of IoT characteristics for future research in this field.

Initially, LSTM outperformed the other algorithms with an accuracy of 78.2%. After adjusting hyperparameters, it reduced misclassification with Glove embedding, and employing other strategies, the final LSTM model was able to predict these cyber-attacks within the IoT environment with a peak accuracy of 87%.

The authors in [63] proposed LSTM-Gauss-NBayes, an anomaly detection technique for large-scale Industrial Internet of Things (IIoT) time-series data generated by millions of heterogeneous sensors. The core idea is that an LSTM-NN can be trained exclusively on normal data, then used to predict future observations based on this training. The difference between actual data and predicted data, known as a time point error, is then fed into a Gaussian Naive Bayes model to classify data points as either normal or abnormal relative to the LSTM-NN forecast.

The method was evaluated using three real-world datasets (Power, Loop Sensor, and Land Sensor) and outperformed competitor models, achieving an average precision of 0.955 and recall of 0.956. In the results for the Power dataset, their reported precision was 0.980 and recall was 0.974. Once abnormal scenarios are identified in the IIoT space through anomaly detection methods, the output can help determine periods of anomalies by highlighting when irregular data might have occurred, either due to an unauthenticated, non-compliant unregistered device or because a registered device has been compromised and is beginning to inject altered data into the entire IoT data system.

The [64] authors introduced DeepAuthen, a deep learning-based framework for continuous user authentication using mobile sensor data. The DeepAuthen framework employs a hybrid approach combining CNN and LSTM architectures to create a DeepConvLSTM model that analyzes activity patterns from accelerometer, gyroscope, and magnetometer data across three benchmark datasets, UCI-HAR, WISDM-HARB, and HMOG.

After filtering, normalization, and segmentation into overlapping time windows, the model employed CNN layers to capture spatial features and LSTM layers to learn temporal dependencies. DeepAuthen achieved state-of-the-art performance, reaching up to 99.99% accuracy and 0.01% EER for some HMOG activities, demonstrating its potential for smartphone user authentication.

Deep learning methods produce significant results for IoT device authentication systems because of their ability to extract advanced features and achieve high accuracy in the authentication processes. Therefore, it is vital to prioritize addressing major challenges, including computational demands, reliance on data, and environmental vulnerabilities.

Table 6 summarizes the studies that reviewed deep learning (DL) approaches for IoT device authentication. These techniques are highly accurate, robust, and scalable across a wide range of IoT contexts. Although these methods exhibit great potential, they have several critical shortcomings including high computational complexity, vulnerability to adversarial attacks, and low efficiency in dynamic or resource-constrained environments. The following discussion breaks these down in terms of their advantages, disadvantages, and security risks.

Research on IoT authentication using 2D-CNN, biLSTM, and 3D-CNN coherent blocks to identify deep temporal patterns (DTPs) showed 96.7% accuracy and high robustness, especially when analyzing 3D-DTP, as well as fast processing across all cases [20]. However, these models are computationally intensive, making them unsuitable for constrained IoT devices with short signal sequences. Moreover, their security is vulnerable due to risks such as spoofing, denial-of-service attacks, and data poisoning in adversarial environments [20]. The deployment of LSTM models for IoT device authentication has increased because they have better model sequences and temporal dependencies than the other models. Their high noise resistance and protocol-agnostic performance enabled them to achieve 99.58% accuracy under LOS (line-of-sight) conditions [65]. Nevertheless, the accuracy dropped to 88% in non-line-of-sight (NLOS) scenarios, highlighting a weakness when the base station is controlled, allowing arbitrary traffic switching by an adversary [65].

In [55], ANNS were studied as a passive authentication measure based on touch dynamics and mental calculations. Mental calculations involve a user performing arithmetic with their registered R-code digits and the P-code digits provided by the server. The user constructs pass-code digits for authentication based on their touch pattern to enter their code. With this method, the false rejection and false acceptance rates (FRRs and FARs) were reduced to 5.03% and 4.36%, respectively, significantly lowering shoulder-surfing risks without additional hardware [55]. However, this approach requires 30–40 login attempts for training, leading to lengthy initial setup times and potential data compromise during the training process [55].

Adaptive ANN models have been demonstrated to adapt dynamically to environmental changes, achieving 100% detection for all SNRs above 6 db and 95% detection for SNRs below 4 db [56]. However, the performance of existing models declines in low SNR conditions, making adaptive ANN models vulnerable to adverse channel conditions and interference [56].

CNN-based models have been widely utilized for RF feature extraction, achieving accuracy comparable to previous state-of-the-art methods, with improvements of at least 10–15% in most cases. CNN models can scale for both small and large IoT networks; however, they require $10^{5}$ samples for training, which entails significant computational cost [53,66,67,68]. Additionally, CNN models are vulnerable to adversarial attacks and privacy issues.

An adversary can compromise authentication results by manipulating the input data [53,66,67,68]. The performance of LSTM-based systems for traffic analysis in time-series has demonstrated usable accuracy (92%) and good sensitivity to changing attack patterns [53,66,68]. However, these LSTM-based systems create about 50–100 ms of latency in real-time scenarios and require repeated training, which diminishes the system’s value. Additionally, they experience a 30% false-negative rate when attempting to detect zero-day attacks, indicating potential vulnerability to poisoning attacks or other types of unknown attacks [53,66,68].

Based on the results of combining anomaly detection with autoencoders for IoT networks, it has been found that state-of-the-art accuracy can be achieved at 95% recall rates with a 10% reduction in false positives compared to traditional techniques [66,67,68]. In contrast, these approaches have large data storage requirements (i.e., >10 GB) and can produce error rates of 15–20% when faced with these changing dynamics. Additionally, the systems were unable to detect more than 60% of zero-day attacks, indicating that they were ineffective against unknown attack scenarios [66,67,68].

DNNs have also been studied for the purpose of multi-device authentication, achieving performance of over 90% accuracy with limited preprocessing methods. DNNs have also been examined for multi-device authentication, achieving accuracy rates over 90% with limited preprocessing techniques [53,66,68]. However, DNNs consume more energy, averaging between 100 and 500 mW, and are particularly susceptible to overfitting when limited feature data is available. Notably, DNN accuracy decreased by 25% during adversarial attacks, further highlighting its limited viability in hostile environments [53,66,68].

RNNs showed 88% accuracy in modeling temporal traffic patterns and were compatible with over 1000 devices [53,67,68]. Conversely, RNNs are prone to gradient-related issues that may limit their convergence or performance, making them unsuitable for low-memory devices. Additionally, RNNs exhibited a 50% zero-day detection error rate, indicating they are not resilient to suggested inputs [53,68].

Federated Learning (FL) decreases privacy risks by 80%, while providing decentralized IoT authentication for over 103 devices [66,67,68]. However, FL encounters latency issues with heterogeneous data (20–50 ms), affecting performance. There is also a risk of data poisoning attacks in FL, which could reduce accuracy by 15% if encryption protocols are not implemented [66,67,68].

CNN-based systems that utilize Channel State Information (CSI) have demonstrated a higher true positive rate (TPR) of 99.64% [69,70]. However, they require 5145 packets for dual-input CNNs and exhibit substantial computational overhead, especially for ResNet50 models, which have $2.5\times 10^{7}$ parameters. Additionally, model accuracy continues to decline as the distance between devices and the number of concurrent users increases [69,70].

Dynamic watermarking and LSTM models have demonstrated promising performance by detecting attacks within 0.1 s and attaining a bit error rate (BER) of 0.001, compared to 0.03 for static watermarking at β/σ=1 [61]. Although this method involves high computation and longer training times, it becomes ineffective if an adversary replicates the signal’s spectral properties [61]. In a CNN-SVM hybrid model with VMD and Tri-Training, 95.01% accuracy was achieved, with a 99.90% success rate for imitation attacks [71]. However, this increases authentication time and battery consumption, making it unsuitable for IoT devices with limited power sources.

Additionally, privacy concerns remain due to a 0.10% success rate of imitation attacks, indicating that further improvements are needed [71]. Finally, ADN models, CNNs, and autoencoders were employed to enhance IoT security, achieving 94.8% accuracy in botnet detection and 99.9% accuracy in fall detection [72]. One limitation of their work is that the model had issues with fading channels and latency in dynamic environments. Moreover, their approach would not be resistant to Trojan-based attacks, which could compromise its effectiveness against complex malware [72].

In Table 6, the research studies presented and organized demonstrate the significant potential of DL approaches for advancing IoT device authentication. However, the aforementioned frameworks face serious challenges due to their high computational costs, adversarial attacks, and poor performance in dynamic environments. To further promote the security and reliability of IoT authentication frameworks, future research should focus on optimizing the computational costs, enhancing adversarial resilience, and improving the ability to detect zero-day attacks.

## 4. Research Gaps in AI-Based Authentication for IoT Devices

The development of secure and efficient IoT device authentication requires identifying research gaps that align with machine learning fundamentals. One major challenge in IoT security is addressing network scalability alongside device resource limitations, as current authentication schemes do not resolve this issue. Furthermore, the dynamic nature of IoT environments and the need for real-time data processing present significant obstacles. Future research should focus on developing authentication solutions that incorporate attack resistance, flexibility, and scalability to ensure secure networking among everyday IoT devices.

### 4.1. Challenges in Machine Learning-Based Authentication

Due to limited computational power, the deployment of machine learning algorithms on IoT devices is difficult, resulting in high resource consumption, intensive computations, and possible privacy issues. Below, we explore all potential machine learning challenges related to IoT device authentication.

#### 4.1.1. Identifying Research Gaps in Current Authentication Approaches

Many existing authentication schemes find it difficult to integrate with the dynamic and heterogeneous nature of IoT ecosystems, and often concentrate on only a limited aspect of machine learning. The key research gaps include the following.

**Lack of Standardized Taxonomies:** According to [28], the inconsistent nature of IoT security taxonomies results in fragmented solution approaches, leading to significant problems. There is a need for a systematic review of authentication and authorization methods, as specific reviews only address particular IoT security threats. Research on battery performance and light computing fails to cover the full range of necessary security needs.

Current research studies focus solely on specialized aspects of IoT security threats while neglecting the changes nodes undergo in IoT networks. The authors in [28] discussed dedicated IoT-specific assaults, such as node capturing and sleep deprivation attacks, highlighting side-channel vulnerabilities that receive isolated treatment instead of being integrated into a comprehensive security design. To achieve suitable IoT applications, a complete and standardized approach to IoT security must be developed through clear taxonomical definitions, alongside the consideration of IoT network dynamism.

**Insufficient Threat Adaptability:** In [73], the authors applied machine learning and deep learning as essential tools for IoT security, as they require adaptive intelligent solutions for real-time threat response. Traditional computing methods are inadequate for addressing the new attack vulnerabilities created by IoT network connections. Protecting IoT systems necessitates the evolution of ML and DL from enabling secure IoT system connections to becoming intelligent security systems.

Enhancing ML and DL models primarily involves three techniques: input preprocessing, improving model resilience, and using malware detection methods. There is a need for evolving security models, as no single defense approach provides complete protection against adversary threats, making ongoing updates necessary. Implementing machine learning models requires anti-spoofing solutions and diverse, extensive datasets, along with real-time processing efficiency and adaptability to changes in physiological and behavioral traits [74].

**Limited Cross-Layer Security:** Research on IoT security concentrates its analysis on individual layers—from perception to communication, and then to data processing and application. However, this approach fails to identify the underlying vulnerabilities that span across different layers, such as man-in-the-middle (MiTM) attacks on MQTT brokers and signature wrapping attacks in cloud servers.

Modern authentication systems implement their protocols across various layers but do not adopt three-way procedural authentication protocols. Research from [20] highlights the need for cross-layer schemes aimed at combating attacks that occur within overlapping IoT architecture layers between IT and OT. The defense system must protect against attacks between layers, including MiTM attacks on MQTT brokers and signature wrapping on cloud servers.

#### 4.1.2. Limitations of Existing Machine Learning Models in IoT Security

The security challenges posed by ML models in the IoT technology are explained below.

**Resource Constraints:** Most IoT devices operate with limited processing power and memory, making it difficult to install standard machine learning models. These devices lack sufficient processing resources to run existing ML methods, particularly in edge computing environments that require power-efficient algorithms. Many traditional IT security tools struggle to integrate with IoT platforms due to specific issues.

The IoT requires specialized security methods that improve both encryption and algorithm efficiency. The most effective way to incorporate ML into IoT security involves finding methods to embed intelligent systems without overloading the device performance. Machine learning offers an ideal framework for adding intelligence to IoT devices, while deep learning excels in predictions; however, ML needs feature engineering and training updates to work effectively in IoT applications [75].

**Data Scarcity and Bias:** Authentication methods in IoT face numerous challenges due to limited information and biased data. Machine learning models require large datasets with diverse entries, but such data are often scarce in IoT settings, which affects the accuracy and predictive performance. Data collected from IoT environments shows irregular collection patterns and unbalanced representations of user behavior, caused by intermittent transmissions and an improper mix of legitimate and malicious requests.

This situation complicates effective training. Biases originating from users, devices, and locations exacerbate authentication challenges, and may lead to unfair outcomes. The successful development of fair ML-based authentication systems relies on using advanced algorithms, data enhancement techniques, bias mitigation methods, and exploring new strategies for learning and circuit design [28].

**Vulnerability to Adversarial Attacks:** In [76], the authors examined the vulnerabilities of deep learning-based IoT device identification through adversarial attacks. Attackers make subtle adjustments to the input data, leading to incorrect predictions with high confidence scores. Such threats can cause significant damage to IoT systems by breaching equipment authentication and compromising device identification, reliable transmission, communication security, and privacy. The signal domain remains vulnerable because of flaws in DL models that perform modulation recognition.

#### 4.1.3. Scalability and Adaptability Concerns in Real-World Implementations

Scalability is a major challenge because IoT networks continue to grow exponentially.

**Dynamic Network Topologies:** The dynamic nature of IoT network topologies poses a significant challenge to effective authentication methods that use machine learning algorithms. Traditional authentication techniques employed in distributed systems, such as 5G or edge computing, require improvements because they struggle to adapt to dynamic adversarial environments.

Authentication processes benefit from machine learning, as it detects temporal characteristics that facilitate secure system adaptation. This enhanced approach incorporates various attributes, such as network selection labels and physical layer specifications, to improve system performance. Machine learning tools assist organizations in utilizing data to create security systems that can scale and operate continuously [77].

**Heterogeneous Device Management:** Managing various devices within IoT systems poses a significant challenge due to the integration of different hardware systems and multiple communication protocols. Currently, ML models struggle to apply generic knowledge across diverse devices, resulting in limited connectivity among them. Security and privacy in IoT networks should depend on traditional cryptographic methods; however, these security solutions are often insufficient for IoT nodes as indicated by [75].

The integration of machine learning and deep learning techniques presents a solution for securing IoT devices and networks through intelligent enhancement of their capabilities. The heterogeneous properties of end devices emphasize a critical requirement for authentication and authorization (AA) schemes as discussed in [28]. Addressing security issues across various IoT devices is possible through the design of heterogeneous AA schemes.

**Latency and Throughput:** Implementing real-time authentication in extensive IoT networks requires an essential evaluation of metrics between latency and throughput, as traditional ML-based designs often overlook handshake duration and end-to-end delay measurements.

In [28], the authors outline several general factors relevant to authentication and authorization (AA) schemes, including average response time, impact on throughput, packet delivery ratio, communication costs, computation costs, and storage/memory costs. Handshake duration evaluates the time needed for the communication setup, whereas the end-to-end delay (E2ED) measures the time taken for data packets to reach their destination. Academic studies face challenges because they use various performance evaluation methods without standardized procedures and overlook the authentication duration standards. The research field should focus on ML-driven AA schemes that incorporate both standard and ML-specific performance metrics to improve security and efficiency in IoT environments.

#### 4.1.4. Addressing Data Privacy and Security Risks in the Authentication System

**Sensitive Data Exposure:** Implementing ML-based authentication in IoT systems forces users to navigate challenges between ensuring security and maintaining privacy, as their biometric and behavioral information becomes vulnerable to attack. When an IoT gateway fails in security protocols, it exposes the decrypted data through conversions from Zigbee to HTTP, making the network highly susceptible to attackers.

Various machine learning and deep learning techniques can be employed alongside authentication protocols for MiTM (man-in-the-middle) defense, as well as nonlinear kernel SVM methods for secure medical data classification. Combining deep learning with intrusion detection systems (IDS) strengthens network security, while end-to-end encryption ensures data confidentiality. Fundamental security measures start with adopting an accepted cybersecurity framework, followed by regular credential updates, network segmentation, threat intelligence monitoring, and ultimately deploying security software to protect IoT systems and their networks. Weak passwords and vulnerabilities in deep learning algorithms must be addressed, as these enhancements will improve the overall security of IoT systems.

**Regulatory Compliance:** Implementing machine learning-based authentication requires fundamental changes because privacy laws, such as GDPR, present challenges when utilizing private data. Two key strategies for data privacy, federated learning and differential privacy, help address the conflicting demands of authentication systems.

The distributed training method in federated learning allows model development through decentralized devices and servers without transferring data, thereby reducing the data concentration needs. Adding noise to the data or model parameters during training under differential privacy ensures that individual data points remain indistinguishable, thus safeguarding users from data disclosure, even from attackers with model access.

The limited application of these strategies in current authentication solutions presents both a challenge and an opportunity, enabling privacy protection without compromising the effectiveness of biometric systems.

**Model Inversion Attacks:** Model Inversion (MI) attacks pose a significant threat to machine learning systems because they allow attackers to extract confidential training information through either attribute inference or data reconstruction. These attacks can be categorized into three access levels: white-box, black-box, and label-only attacks, with white-box attacks being the most perilous due to their provision of complete model access.

The various inference and reconstruction methods employed in MI attacks allow for a distinction between attribute deduction via inference attacks and data reconstruction. The risk escalates notably in systems that manage the real-time processing of sensitive information, such as continuous authentication systems. Several defense strategies should be adopted to protect against these attacks, including differential privacy, input/output masking, secure multi-party computation, and federated learning [78].

### 4.2. Comparative Analysis of IoT Authentication by Machine Learning and Traditional IoT Device Authentication Methods

The traditional method of authenticating IoT devices relies on static credentials such as passwords and pre-shared keys. Standard authentication practices for IoT devices remain vulnerable to attacks utilizing brute force methods and credential theft. Integrating machine learning enables the development of adaptive authentication systems that adjust to environmental contexts. System analysis via ML algorithms facilitates the identification of live security threats by employing three types of data: device behavior patterns, network activities, and user performance. The system enhances security through a dynamic mechanism that allows it to learn and adapt to emerging threats.

The next part of this analysis explores these areas. According to research on [39,79,80], traditional methods often depend on password-based systems, cryptographic techniques, and hardware-based solutions. However, the machine learning mechanisms for IoT device authentication use algorithms such as SVM, DT, RF, and deep learning models like CNN, RNN, and LSTM for anomaly detection and threat identification. A comprehensive analysis of both authentication approaches involves examining several key dimensions that include the following:

- **Scalability:**

Typical security frameworks in traditional methods struggle at a large scale due to the need for manual intervention, which limits their operational capabilities. However, device fingerprinting combined with behavioral biometrics in machine learning systems provides exceptional scalability, as it can automatically maintain security for numerous devices.

- **Resource Efficiency:**

Low-power IoT devices encounter challenges because of the resource-intensive nature of PKI authentication. Nevertheless, machine learning algorithms can be designed to function efficiently, which makes them suitable for various IoT devices.

- **User Experience:**

Behavioral biometrics, which provide continuous user authentication through machine learning, allow users to enjoy seamless experiences without interruptions to their work. While control losses are minimized, negative user experiences may still arise through two-factor authentication (2FA) methods. This analysis focuses on the key advantages and drawbacks of two authentication methods.

**Weaknesses:**

Traditional authentication methods face three significant weaknesses: vulnerability to brute-force attacks, password theft, and limited resources available. Machine learning approaches require human assistance to function and lack sufficient flexibility for growth. Their primary limitations include the need for extensive training data, lengthy computational processing, additional challenges related to data privacy, and the potential for model bias.

**Benefits:**

Traditional security provision involves utilizing existing infrastructure systems and implementing established security practices. Enhanced security and real-time anomaly detection can be achieved through machine learning techniques, which also provide scalability, improve the user experience, and automate the identification of new threats. In the table below, detailed comparisons of both are shown.

Table 7 shows a comparative evaluation of traditional authentication methods and those using machine learning (ML) for IoT systems, evaluating both similarities and differences across several aspects. We assess them through key dimensions, namely authentication approach, security aspect, vulnerability to attack, scalability, adaptation to threats, latency, energy consumption, maintenance, cost, and integration with IoT devices.

The analysis reveals that while both approaches fundamentally share the objective of securing IoT devices from unauthorized access, the methods and impacts vary significantly. Traditional authentication mechanisms (PKI, passwords, digital certificates, biometrics, IP/MAC addresses, tokens, and MFA) utilize static credentials that are useful, but if they are not updated frequently, they are susceptible to compromise.

In contrast, ML-based approaches (anomaly detection, behavior profiles, and continuous monitoring) use dynamic techniques to identify when a device behaves differently from its expected behavior, thus increasing security in response to changes in the threat landscape. Security mechanisms associated with traditional solutions vary from low assurance (e.g., passwords and PSK) to high assurance (e.g., digital certificates and MFA) but are generally vulnerable to brute-force attacks and credential theft [82,83].

Alternatively, methods based on ML employ continuous learning and pattern recognition to detect abnormal actions in real time and provide better protection against emerging cyber threats [84]. In terms of vulnerability to cyber-attacks, static credentialing approaches are susceptible to credential theft and brute-force attacks simply because they rely on static credentials [56,85]. In contrast, machine learning (ML)-based models are inherently more robust, as they continuously learn from data and are consequently capable of identifying and mitigating newly emerging cyber threats. Scalability is another major issue that poses challenges for traditional methods, primarily because of the need for manual credential management and configuration of each device [38,86].

ML-based methods enable automatic updates and the real-time detection of new anomalies, making them highly scalable for large-scale IoT environments. In conventional techniques, when addressing new threats, the traditional approach relies on manual updates (e.g., updating passwords and renewing certificates), which adds to the administrative burden [56]. In contrast, systems based on ML utilize continuous learning and data-rich information to automatically adjust to evolving threats, thereby minimizing human engagement [87].

Latency performance is another area in which ML-based systems excel. Certificate validation and MFA methods, for example, incur high latency due to their reliance on cryptographic operations [56]. For ML models optimized for edge computing and real-time anomaly detection, they eliminate latency caused by cryptographic operations, enabling quicker processing and decision-making processes [88]. In relation to energy consumption, traditional methods, particularly digital certificates and MFA, are highly resource intensive and consume a significant amount of energy, which can be problematic for resource-limited IoT devices [89].

Conversely, ML-based systems can ease resource limitations by using low-power learning algorithms. Traditional maintenance involves extensive manual updates, which increases operational complexity. In contrast, ML techniques continue to learn and adapt, reduce maintenance requirements, and boost security with minimal or no manual effort [90]. The financial analysis indicates that traditional authentication methods are low cost initially; however, they incur higher operational costs because of credential management and updates [91].

In contrast, ML-based systems have higher initial costs for training the model but very low operational expenses in the long run because of automated monitoring and anomaly detection [92]. Finally, while traditional approaches are easier to implement for IoT devices, especially fundamental methods such as PSKs and passwords, the simplicity of implementation becomes more complicated when considering recent approaches such as multi-factor authentication [93]. ML-based methods do not require additional complexity for integration with IoT devices because they use edge computing and continuous learning for seamless integration [38].

## 5. Lessons Learned and Open Challenges

### 5.1. Research Challenges

**Lack of Real-World Datasets**

The datasets currently in use do not adequately represent the diverse operational environments of IoT devices under dynamic conditions. As a result, developing and testing resilient IoT applications is challenging. For example, deploying deep learning models in IoT necessitates access to large datasets from edge locations, as these models require substantial real-world data. Research aimed at detecting performance anomalies in edge computing relies on publicly available edge datasets, which are currently lacking in the public domain. The creation of extensive datasets has become increasingly complicated due to the intricacies of IoT time series data, which display various spatial and temporal patterns [94].

**Adversarial Vulnerabilities**

Adversarial attacks on the ML used in IoT systems aim to deceive the models with malicious inputs. Security and reliability in IoT applications are compromised by adversarial vulnerabilities. Research shows that deep models are especially vulnerable to various adversarial attacks, and the existing defense mechanisms are inadequate. Systematic research demonstrates the need to develop effective defense mechanisms to ensure security in ML models. Adversarial ML presents challenges in identifying the specific goals of attacks, crafting effective solutions, and understanding the causes of adversarial vulnerabilities.

**Integration Constraints**

The implementation of ML-based systems within IoT networks faces challenges related to data management, complex computations, and privacy issues. The integration of ML and AI into IoT systems necessitates solutions for three primary challenges: ensuring data management accuracy, cloud computing security, and blockchain security. To address these objectives, edge computing combined with federated learning and distributed intelligent systems must be employed, as they help overcome existing barriers in IoT systems. These strategies enhance the efficiency and security of IoT systems by positioning data processing and computational tasks closer to their data sources.

### 5.2. Future Directions

**Advanced Machine Learning and Deep Learning for IoT Authentication**

Modern IoT networks demand sophisticated authentication methods due to their increasing complexity. Advanced analytics, including ML and DL, can effectively detect and address vulnerabilities through real-time communications. Future research should focus on creating lightweight and scalable ML/DL models tailored to IoT requirements such as limited computational capacity and energy efficiency. Additionally, deep learning models must be resilient against adversarial attacks and secure in dynamic IoT environments.

**Reinforcement Learning for Adaptive Security Policies**

The security policies of IoT devices can achieve optimal performance levels through RL, as these devices learn through ongoing interactions with their environment. Authentication methods built with RL do not rely on static dataset information; instead, these mechanisms can respond dynamically to device behavior, network conditions, and emerging threats. The study of reinforcement learning techniques for optimizing authentication strategies requires further investigation, particularly for extensive and resource-limited IoT systems. Combining multi-agent RL solutions can enhance cooperative authentication methods and strengthen security against complex cyber-attacks.

**Adversarial Robustness and Secure Model Training**

Authentication systems utilizing ML are vulnerable to adversarial attacks, as attackers can manipulate input data to bypass security protocols. Future research could enhance the resilience of ML/DL authentication systems by developing adversarial training, robust feature extraction, and anomaly detection methods. Additionally, creating new defensive countermeasures, such as uncertainty-aware learning and automated adversarial detection, will be crucial for ensuring the reliability of authentication in high-threat IoT environments.

**Standardized Benchmarks and Model Evaluation**

The lack of standardized datasets and evaluation metrics for IoT authentication hinders the reproducibility and replication of the research findings. Standardized benchmarks can enable the fair assessment of different authentication technologies and accelerate advancements in the field. Future efforts should focus on establishing open-access datasets, clearly defined performance metrics, and a comprehensive testing protocol to ensure reliability and scalability of authentication solutions.

**Ethical Considerations and Regulatory Compliance**

The privacy, data ownership, and algorithmic bias related to the increasingly advanced use of IoT authentication mechanisms must be addressed. Future research should ensure that authentication techniques comply with regulatory frameworks such as the GDPR and HIPAA. Furthermore, it is essential to study user acceptance and trust in ML/DL-based authentication to develop security solutions that are both effective and ethically responsible.

## 6. Conclusions

This paper thoroughly reviews ML-based authentication protocols, their methods, and implementation challenges. Active ML-based authentication systems will become increasingly vital as the number of networked devices grows. Device authentication has made significant progress through the integration of ML, deep learning, and reinforcement learning.

The evolution of device authentication via machine learning introduces innovative solutions that address issues of scalability and security vulnerabilities. By employing ML techniques, systems can effectively detect abnormal behaviors. Implementing a deep learning framework to support multi-layer neural networks enables users to learn and extract important feature sets from data with different dimensions, thereby creating scalable authentication models.

These models contain advanced features for securely verifying identities across various platforms. Deep learning technology allows the development of robust and reliable authentication systems that protect data, making it difficult for unauthorized individuals to gain access. Reinforcement learning offers IoT applications a proactive approach to optimize authentication methods based on changing environmental conditions, especially when resources are limited.

Using techniques from machine learning (ML), deep learning (DL), and reinforcement learning (RL) creates a layered defense system that manages persistent threats while improving threat assessment. This three-method approach enhances resilience against sophisticated attacks on IoT ecosystems, and can only improve the safety of IoT environments. However, implementing these methods in practice faces considerable challenges. High computational requirements, extensive training durations, and the difficulty of using casually collected large-scale datasets significantly hinder their effectiveness. Although RL provides an alternative by learning in a customized manner, it struggles to converge from optimal states as policies change substantially over time. DL models tend to be accurate but often lack interpretability and are vulnerable to attacks that exploit their weaknesses through adversarial tactics. Many of these issues are worsened by data limitations, such as difficulties in obtaining relevant datasets and labeled data for rare but critical attacks like Advanced Persistent Threats (APTs). There is also a heightened privacy risk when ML-based approaches handle sensitive, identifiable, and sometimes harmful behavior data and attempts.

Besides technical limitations, resource constraints in IoT devices worsen these issues. Computing power, memory capacity, and energy availability limit the ability of resource-intensive models, such as ML or DL, to operate on local devices. Cloud offloading can ease some of these burdens, but introduces additional challenges by causing latencies and creating single points of failure. Furthermore, with large-scale deployments, deploying across diverse devices and enabling continual learning to address new threats, such as zero-day vulnerabilities. Currently, static models cannot be easily adapted to ongoing learning.

Future research could focus on developing lightweight algorithms specifically for Iot devices and federated learning based on privacy-preserving training and lifelong learning, enabling IoT users to adapt in dynamic environments (i.e., adjusting their authentication). New standardized datasets and benchmarks are essential for fair evaluation and comparison of solutions. Finally, hybrid edge–cloud architectures would support energy-efficient methods and offer the best compromise to develop secure, scalable, and practical IoT authentication techniques.

## Figures and Tables

**Table 1:** Security risks and mitigation across IoT components.

Component	Vulnerabilities	Assessment Tools	Challenges	Security Measures
Software [13,14]	Insecure APIs, encryption flaws, injection, firmware bugs, buffer overflows, MITM, DoS, remote code exec [13,14]	Firmadyne, DiscovRE, IoTFuzzer, manual RE, security frameworks [13,14]	Limited resources, device variety, lack of standards, firmware access [13,14]	Secure coding, firmware analysis, updates, authentication, patching, monitoring [13,14]
Hardware [15,16,17]	Default credentials, outdated TCP/IP stacks, open ports, reused keys [16,17]	Shodan, Nessus [17], NIST 800-22 [15]	Low memory, protocol diversity (CoAP, MQTT), device constraints [17]	Secure boot, disable ports, tamper resistance, lightweight encryption [15,16,17]

**Table 2:** Comparison of IoT device authentication types.

Type	Verification Process	Credentials	Vulnerabilities	Use Case	Technologies
Static [8,9,18]	One-time, fixed checks [8]	Passwords, keys, MD5, certs [8,18]	Brute force, phishing, replay, key theft [8,18]	Low-security or legacy IoT [9,18]	MD5, AES, RSA, static certs [9]
Dynamic [10,11,19,20,21]	Context or behavior based [21]	RF prints, keystrokes, sensors [20]	Noise, impersonation, replay, memory limits [10,11]	High-security, adaptive IoT [21]	LSTM, RNN, biometrics, PUFs [10,11]

**Table 3:** Supervised ML for IoT device authentication.

Methods	Benefits	Drawbacks	Security Issues
Support Vector Machines (SVMs)	High accuracy (97.1%) [32] Effective with 10K+ samples [32]	Long training time: 5–10 min (1 K samples), 2h (10 K samples) [32] Limited effectiveness with fewer than 1 K samples [32]	High false rejection rate (50%) in low-data settings [32,34] Vulnerable to adversarial attacks [28,32,34]
Random Forest (RF)	High accuracy across datasets: 98.1% (IoT authentication) [37], 99% (BoT-IoT) [35]	Large memory requirement (16 GB RAM for 30 K rows × 500 features × 1 K trees) [32] Fails with extensive datasets (400 K rows × 50 trees) [32,38]	Susceptible to adversarial input, DoS, and training contamination [31,35,37]
K-Nearest Neighbors (KNNs)	Effective accuracy: 74.58% (keystroke analysis) [41] Low error rate (EER = 2.52% for known users) [32]	Large dataset requirement (712 K+ keypresses) [34] High false rejection rate (FRR = 25%) with noisy input [41]	False acceptance rate (FAR = 10–15%) for impostors [41] Privacy concerns due to user variability [32,34]
Naïve Bayes (NB)	Varied accuracy: 64–99% across datasets [31,34,35]	Assumes feature independence, impacting real-world performance [35]	Susceptible to misclassification and dataset poisoning [32,35]
Decision Trees	High accuracy (96.32% for IoT smart home) [31] Reliable across datasets (99% for BoT-IoT) [35]	Varied precision range (0.97–0.98, XGBoost performs better) [35] Performance declines with limited data (2–8 devices, 483 flows) [31]	Keystroke exposure risks [34] Traffic manipulation vulnerabilities [35]
Logistic Regression	Applicable to small datasets (483 traffic flows) [31] Suitable for IoT (2–8 devices, Raspberry Pi) [31]	Lower accuracy than Decision Trees (96.32%) [31] Limited predictive flexibility due to linear assumptions [31]	Susceptible to packet manipulation [31] False positives: 5, False negatives: 12 [31]
Linear Regression	High real-time accuracy (98.07%) with minimal delay (<3 s) [42] Effective billing prediction (91.98%, RMSE 0.0493) [42]	Reliable variable prediction (e.g., 89.48% in Room 10A, RMSE 0.0596) [42] Limited granularity (day-based intervals, lacks hourly/dynamic options) [42]	Security risks: Weak/default passwords on PZEM-004T, NodeMCU, APIs [42]

**Table 4:** Unsupervised ML methods for IoT device authentication.

ML Method	Benefits	Drawbacks	Security Issues
K-means Clustering [41,46]	Acc. ↑ from purity 0.44 to 0.877 with preprocessing; clusters efficiently (2–50 clusters)	Noise-sensitive; init. purity = 0.44 (keystroke); requires preset *k*	DoS on IoT; false auth. due to noise
PCA (Principal Comp. Analysis) [41,45]	Reduces dimension; speeds up processing; useful in CSI-based keystroke inference (64–82% acc.)	Poor clustering: purity = 0.20, neg. silhouette; weak on fine-grain CSI	Vulnerable to data perturbation; usable for PIN inference attacks (64–82%)

**Table 5:** Reinforcement learning (RL) approaches for IoT device authentication.

Approach	Pros	Cons	Security Issues
Auth. via RL for Risk Adaptation [49]	G-Mean = 92.62%; dynamic challenge adj. via DES-DRL; trained every 1000 obs.	130 h offline training; 6000 samples (∼1 week) for convergence; high memory	Class imbalance (bf = 0.25); vulnerable to context-based misuse; on-device privacy preserved
RL for IoT Interface Control [47]	Learns opt. seq.: Goal 1 (2 steps), Goal 2 (4 steps); 400 interactions; finds alternates (e.g., dim = off)	Goal 2 slow (>100 episodes); 40 min per 100 episodes due to 250–600 commands	Learned FSMs may be exploited via undocumented protocols; weak interop. creates risks
DL + RL for IoT Auth. [50]	Handles heterogeneous data; scalable with deep models	Limited real-world validation; no detailed acc. metrics	Modified inputs can cause auth. failure; DoS attacks degrade system integrity
Adaptive ϵ-Greedy RL for Security [51]	ϵ adjusted (0.1–0.9) by attack freq.; PDR = 1.0 (benign), 0.929 (malicious) @ 160 units	Delay: 1489 ms (malicious), 1178 ms (non-malicious); slower in attack scenarios	Proxy user mimicry; black hole attacks drop packets; limited resources increase risk
RL + ECC for Auth. [52]	ECC base *G* resists insider attacks; XORed nonces $r_{1}/r_{2}$ ensure confidentiality; no plaintext shared	>72 h for 1000 users (Jupyter); α = 0.1–0.5, γ = 0.6–0.9; slow for constrained devices	Without nonces: MITM risk; ECC/LDAP failure exposes spoofing/ replay vulnerabilities

**Table 6:** DL methods for IoT device authentication.

DL Method	Benefits	Drawbacks	Security Issues
2D-CNN, 3D-CNN + biLSTM [20]	96.7% accuracy; good for 3D-DTPs; efficient computation	High resource use; limited with short signals	Susceptible to spoofing, DoS, poisoning
LSTM for Auth. [65]	99.58% in LOS; works in noise; protocol-free	Drops to 88% in NLOS; overfitting possible	Base station compromise risk
ANN (Touch Dynamics) [55]	FRR 5.03%, FAR 4.36%; no extra HW	Needs 30–40 logins to train	Training data may be leaked
Adaptive ANN [56]	100% detect. @ SNR ≥ 6 dB; robust @ 4 dB	Drops in low SNR	Susceptible to interference
CNNs (RF Features) [53,66,67]	+10–15% accuracy; tunable; scalable	Needs $10^{5}$ samples for HPC	Prone to adversarial/privacy attacks
LSTMs (Traffic Analysis) [53,66,68]	2% gain; adapts well to attacks	50–100 ms latency; needs retraining	30% false negatives (zero-day), poisoning risk
Autoencoders (Anomaly Detect.) [66,67,68]	95% recall; 10% fewer false positives	15–20% error with >10 GB data	Poor zero-day detect., false data vulnerable
DNNs (Multi-Device) [53,66,68]	90% accuracy; low preprocessing	100–500 mW energy; overfitting risk	−25% acc. due to adversarial attacks, privacy threats
RNNs (Traffic Modeling) [53,67,68]	88% for 1 K devices; scalable	Gradient issues on low-RAM devices	50% miss rate (zero-day); input manipulation
Federated Learning [66,67,68]	−80% privacy risk; supports 1 K devices	20–50 ms latency with heterogeneity	Poisoning cuts acc. 15%; risk of data leaks
CNN-CSI [69,70]	99.64% accuracy; high TPR	Needs 5145 packets; ResNet50 = $2.5\times 10^{7}$ params	Acc. drops with user separation
LSTM + Watermarking [61]	0.1 s detect. time; BER = 0.001 vs. 0.03	Long training; high complexity	Fails if attacker mimics spectral traits
Hybrid CNN-SVM + VMD [71]	95.01% acc.; 99.9% imitation resist.	High battery use; slow auth.	0.1% imitation breach leaks privacy
ADN/CNN/Autoencoder [72]	94.8% botnet, 99.9% fall detect.	Lower acc. in fading channels	Trojan detect. unreliable under latency

**Table 7:** Comparison of traditional vs. machine learning-based IoT authentication.

Aspect	Traditional Methods	ML-Based Methods	Similarity	Difference
Auth. Mechanism [81]	Passwords, PKI, MAC/IP, MFA	Behavioral patterns, anomaly detection	Both secure IoT access	Static credentials vs. dynamic profiling
Security Features [82,83,84]	Low to high (PSK to MFA)	Real-time threat detection, learning models	Strong security goals	Manual strength vs. adaptive response
Attack Vulnerability [56,85]	Spoofing, brute force, theft	Resistant to new/unseen attacks	Access control against threats	Static failure vs. adaptive resilience
Scalability [38,86]	Manual setup limits scale	Auto-model updates, online learning	Scale with IoT growth	Manual vs. autonomous scalability
Threat Adaptation [56,87]	Manual updates needed	Continuously adapts to attacks	Evolves with threat landscape	Reactive vs. proactive learning
Latency [56,88]	High due to crypto/MFA ops	Low with optimized inference	Impacts user access time	Traditional slower than ML
Energy Use [86,89]	High for certs/MFA	Efficient edge models	Energy-constrained IoT relevance	Higher traditional consumption
Maintenance [85,90]	Frequent manual updates	Minimal updates, self-adaptive	Ongoing system upkeep	Traditional needs more manual work
Cost [91,92]	Low setup, high upkeep	High setup, low upkeep	Resource investment trade-off	Traditional cheaper upfront
IoT Integration [38,93]	Easy for simple devices	Needs infrastructure, compute	IoT device compatibility	Traditional fits constrained IoT

## Data Availability

Not applicable.
